# Transient dormant monomer states for supramolecular polymers with low dispersity

**DOI:** 10.1038/s41467-020-17799-w

**Published:** 2020-08-07

**Authors:** Krishnendu Jalani, Anjali Devi Das, Ranjan Sasmal, Sarit S. Agasti, Subi J. George

**Affiliations:** 1 Supramolecular Chemistry Laboratory, New Chemistry Unit, School of Advanced Materials (SAMat), Jawaharlal Nehru Centre for Advanced Scientific Research (JNCASR), Jakkur, Bangalore, 560064 India; 2 New Chemistry Unit & Chemistry & Physics of Materials Unit, School of Advanced Materials (SAMat), Jawaharlal Nehru Centre for Advanced Scientific Research (JNCASR), Bangalore, Karnataka 560064 India

**Keywords:** Self-assembly, Supramolecular polymers

## Abstract

Temporally controlled cooperative and living supramolecular polymerization by the buffered release of monomers has been recently introduced as an important concept towards obtaining monodisperse and multicomponent self-assembled materials. In synthetic, dynamic supramolecular polymers, this requires efficient design strategies for the dormant, inactive states of the monomers to kinetically retard the otherwise spontaneous nucleation process. However, a generalized design principle for the dormant monomer states to expand the scope of precision supramolecular polymers has not been established yet, due to the enormous differences in the mechanism, energetic parameters of self-assembly and monomer exchange dynamics of the diverse class of supramolecular polymers. Here we report the concept of transient dormant states of monomers generated by redox reactions as a predictive general design to achieve monodisperse supramolecular polymers of electronically active, chromophoric or donor-acceptor, monomers. The concept has been demonstrated with charge-transfer supramolecular polymers with an alternating donor-acceptor sequence.

## Abstract (web summary)

Monodisperse and well-defined self-assembled materials can be obtained by fuel-driven temporally controlled supramolecular polymerization via the buffered release of monomers. Here the authors show that a redox-responsive transient dormant state of monomer generated by redox reaction can lead to supramolecular polymers with low dispersity.

## Introduction

One of the current, grand challenge in the field of supramolecular polymerization is to attain monodisperse structures with precise structural and sequence control^1–5^. Inspired by the covalent organic polymerization, living supramolecular polymerization has been recently shown as a promising strategy for monodisperse supramolecular assemblies^6,7^. Seminal works of Meijer and coworkers on the mechanistic investigation^8–13^ and on the understanding of complex energy landscape of supramolecular polymerization process^14,15^ has been very influential to realize this structural control. Living supramolecular polymerization of small monomeric molecules has been achieved by the elegant design of dormant monomer states for the buffered release of monomers, which in turn facilitated a controlled growth, by kinetically retarding the independent nucleation process. Sugiyasu, Takeuchi, and coworkers in their pivotal work have shown that metastable states formed via off-nucleation pathway process under kinetic conditions, can be used as efficient dormant state for the monomers for the controlled nucleation-growth supramolecular polymerization^16–19^. A similar strategy has been recently extended to many π-conjugated monomers, where seeded and cooperative growth were exploited for obtaining assemblies with precise degree of polymerization^20–28^. In another independent and pioneering strategy, Aida and coworkers have reported a chain-growth supramolecular polymerization for monodisperse structures, by the design of conformationally dormant bowl-shaped monomers with pre-organized intramolecular hydrogen bonding and corresponding N-methylated derivatives as initiator molecule with facially unsymmetric hydrogen bonding features^29^. Our group has recently introduced a bio-inspired, fuel-driven cooperative and living supramolecular polymerization approach by the design of stable dormant states, which can be grown under kinetic control by the binding of small molecules like ATP or a chemical reaction^30,31^. These dormant states for the monomers remain in their inactive form unless triggered by a fuel and hence the rate of fuel generation or its binding action determines the kinetics of growth. Other design strategies have also been reported for the structural control of different kinds of functional supramolecular polymers^32–40^. While the above examples show the importance of the temporally controlled living systems, these strategies to achieve monodisperse structures remain to be system and molecular structure specific as the properties such as mechanism, energetic parameters, and monomer exchange dynamics significantly varies among various classes of supramolecular polymers. Thus, designing a generalized strategy for dormant monomer states to accommodate diverse structural features into the controlled supramolecular polymerization remains to be a desirable attribute.

In this context, transient-dormant states can be considered as a generalized design for the electronically active monomers, wherein a redox reaction converts the active monomers into an inactive transient redox state and the subsequent temporal oxidation/reduction releases the active monomers for a kinetically controlled growth. It provides an alternative chemical approach to achieve high-energy dormant monomer states for highly dynamic supramolecular polymers, where kinetically formed metastable dormant states^16,17^ are inaccessible due to fast monomer exchange dynamics. Although, redox-transient states of the monomers has been recently used for the design of out-of-equilibrium supramolecular assemblies, they are not yet exploited as a dormant monomer reservoir for temporally controlled supramolecular polymerization by the buffered release of monomers^41–45^. Further, such a supramolecular polymerization strategy would be analogous to the well-known reversible-deactivation radical polymerization methods for the synthesis of living chain-growth covalent polymers with narrow dispersity^46^.

Charge-transfer (CT) supramolecular polymers having redox-responsive, donor-acceptor monomer sequence are an ideal class of materials to validate the concept of redox-responsive transient-dormant states. CT supramolecular co-polymers, with alternating sequence of donor and acceptor monomers are important functional nanostructures for supramolecular electronics and ferroelectrics^47–52^. However, despite diverse potential applications, the structural and temporal control in these nanostructures still remain a challenge, which is essential to fabricate them for various functional outcomes. Previously reported metastable or intramolecular H-bonded dormant state designs for the living supramolecular polymerization cannot be extended for CT based systems, as they lack in hydrogen bonds and are highly dynamic to be trapped in high-energy metastable dormant states^16,17^.

Herein we introduce the concept of transient-dormant states for the structural control of CT derived supramolecular polymers. We show the precision supramolecular polymerization of an amphiphilic, donor-acceptor monomer to yield monodisperse CT supramolecular polymers (polydispersity index (PDI) ~1.03) with high degree of polymerization (2–3 μm in length), using redox mediated transient states of the monomers. The monomer, which is a foldameric amphiphile, in its neutral state exists in a folded state which is prone to spontaneous growth, whereas on reduction it unfolds to an inactive dormant state. Remarkably, reversible generation of active monomers by a controlled oxidation process leads to the dispersity control. The dispersity and molecular weight of these supramolecular polymers are highly dependent on its growth kinetics, which can be modulated by controlling the stability of dormant states and oxidation rates by varying concentration of reducing agent.

## Results

### System design

**Fig. 1:**
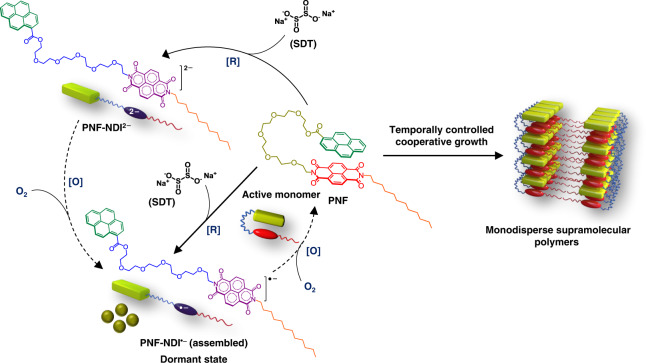
Monomer design. Molecular structure of PNF charge-transfer foldamer and the schematic representation of its amphiphilic assembly into one-dimensional supramolecular polymers by lamellar organization of the bilayers. Kinetically controlled pathway of PNF assembly involves the generation of unfolded PNF-NDI^•−^ and PNF-NDI^2−^ as dormant states for the monomers using sodium dithionite (SDT) as reducing agent and its subsequent temporal oxidation reversibly regenerate the active PNF foldamers in a controlled manner that undergo a kinetically controlled growth. [R] and [O] represents reduction and oxidation processes, respectively. Temporally controlled growth of PNF via redox-dormant pathways results in highly monodisperse supramolecular polymers.

In this work, we have designed an amphiphilic intramolecular CT foldamer (PNF) bearing pyrene as a donor component covalently attached to a naphthalenediimide (NDI) acceptor component, via flexible hexaethylene glycol linker. PNF exist in a foldameric conformation, by virtue of strong intramolecular CT interactions between pyrene and NDI chromophores and these folded amphiphiles self-assemble into one-dimensional (1-D) supramolecular polymers with stacked bilayers. We further show a redox-responsive conformational switching of PNF to an unfolded state, by the reduction of the NDI to its radical anion or dianion form, thereby destroying the intramolecular CT interactions (Fig. 1). The reduced, unfolded PNF-NDI^•−^ state exist in a self-assembled state due to the interactions between NDI radical anions and hence can be considered as a transient assembly which would oxidize back to the folded monomers in a temporal manner. Hence we have used the unfolded conformational state of the PNF as a dormant state for the monomers and its controlled oxidation to active folded monomers results in the kinetically controlled growth of CT supramolecular polymers.

**Fig. 2:**
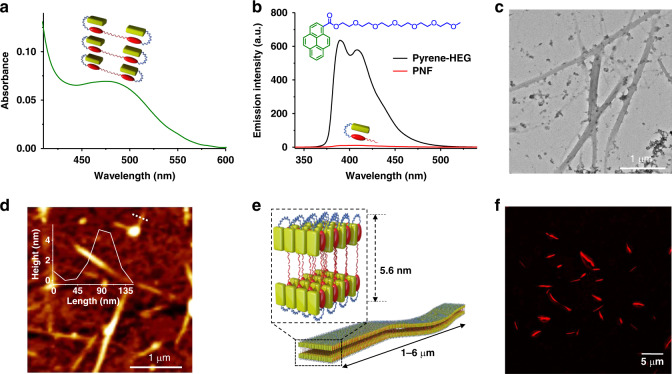
Characterization of the native folded conformation of PNF and its self-assembly. **a** CT absorption band of PNF, originating from the intramolecular folded conformation and **b** corresponding quenched emission of pyrene due to CT interaction, compared with the emission of the model molecule, Pyrene-HEG, (*λ*_exc_ = 300 nm). Inset shows the molecular structure of Pyrene-HEG. **c** TEM and **d** AFM images of the tape structures along with the height profile shown in inset. **e** Schematic for the bilayer packing of the folded PNF molecules in the tape structure. **f** Structured illumination microscopy (SIM) image of the tape structures loaded with Nile red dye in the bilayer (*λ*_exc_ = 561 nm). [PNF] = 5 × 10^−5^ M, [Pyrene-HEG] = 5 × 10^−5^ M, [Nile red] = 5 µM. CH_3_CN/H_2_O (1:1 v/v), pH = 8, buffer.

The PNF foldamer was synthesized according to the following procedure. First, hydroxyl terminated NDI **(3)** was synthesized via statistical imidation reaction (See Supplementary Fig. 1) of naphthalenetetracarboxylic dianhydride (**1**) with dodecyl amine and hexaethylene glycol amine (**2**, See Supplementary Fig. 2). Subsequent EDC esterification reaction between the molecule **3** and 1-pyrene carboxylic acid (**7**) yielded the product PNF, (See Supplementary Fig. 3) which was thoroughly characterized by various spectroscopic technique. A control molecule Pyrene-HEG (inset, Fig. 2b) was also synthesized for comparing the spectroscopic properties (See Supplementary Fig. 4). The synthetic details and characterization data of all molecules are reported in the Supplementary Methods.

### Self-assembly of PNF CT-amphiphile

First we investigated the self-assembly characteristics of the PNF foldamer (c = 5 × 10^−5^ M) in acetonitrile (CH_3_CN) and water (H_2_O) solvent mixture. Solvent composition dependent study shows that the PNF exist as molecularly dispersed state in pure acetonitrile and as assembled state in acetonitrile-water (1:1 v/v) solvent mixture, as evident from the red-shifted π to π* absorption band (~5 nm) of NDI chromophore in the PNF foldamer and appearance of scattering (See Supplementary Fig. 5). The CT interaction between pyrene and NDI chromophores in its assembled state is evident from the characteristic red-shifted broad CT absorption band at 485 nm and the quenched emission compared to the model Pyrene-HEG molecule (Fig. 2a, b). Concentration dependent studies on PNF assembly showed a linear decrease in the CT absorbance at 485 nm with dilution, characteristic of intramolecular interactions and thus indicative of a folded native conformation of PNF in solution (See Supplementary Fig. 6). PNF monomer in its folded conformation is an amphiphilic structure with a packing fraction of 1.05 and hence expected to grow in a lamellar organization (See Supplementary Fig. 7). Accordingly, dynamic light scattering (DLS) measurements of the folded PNF in CH_3_CN/H_2_O (1:1 v/v) suggest that it indeed assembles in solution (See Supplementary Fig. 8). Further, the morphological investigations of the resultant self-assembly using transmission electron microscopy (TEM) (stained using 0.1 % w/v uranyl acetate in water) and atomic force microscopy (AFM) revealed the formation of 1-D tape-like structures with several micrometer length and an average width of 100 nm (Fig. 2c, d and See Supplementary Fig. 9). Further, AFM height analysis of these tapes showed a minimum height of 5 nm, which matches well with the calculated length (5.8 nm) of a bilayer of folded PNF molecules (See Supplementary Fig. 10). Further thin-film XRD pattern of dried samples of PNF structures showed a maximum d-spacing of 58.4 Å along with the reflection peaks corresponding to 28.65 and 18.94 Å, in 1:1/2:1/3 order, characteristic of an ordered lamellar organization of these PNF bilayers (See Supplementary Fig. 11). Based on these observations, we propose that the tapes are formed by the vertically stacked bilayers of folded PNF molecules, with respect to the growth direction as shown in the schematic (Fig. 2e). In an attempt to visualize these tape structures in solution phase without any solvent drying effects, hydrophobic Nile red dye loaded solution of PNF was imaged using structured illumination microscopy (SIM) and confocal laser scanning microscopy (CLSM). Nile red dye is known to exhibit quenched emission (*λ*_max_ at 660 nm) in polar aqueous media and an enhanced blue shifted emission (*λ*_max_ at 630 nm) when encapsulated into the hydrophobic environment of bilayers^53^. Loading of an external dye marker is required to image under fluorescence microscopy as both pyrene and NDI fluorescence is completely quenched in these CT assemblies. The ordered bilayer packing in PNF assemblies allowed an efficient and uniform encapsulation of Nile red dye and hence the 1-D assemblies could be clearly visualized under SIM (*λ*_exc_ = 561 nm, Fig. 2f). Self-assembly of PNF foldameric monomers to the 1-D tapes is very fast resulting in uncontrolled polydisperse assemblies (See Supplementary Fig. 12) and hence a kinetically controlled growth of PNF molecules via a redox-active, unfolded dormant state was further attempted to modulate the dispersity and degree of polymerization.

### Reduction induced disassembly of PNF 1-D tapes

**Fig. 3:**
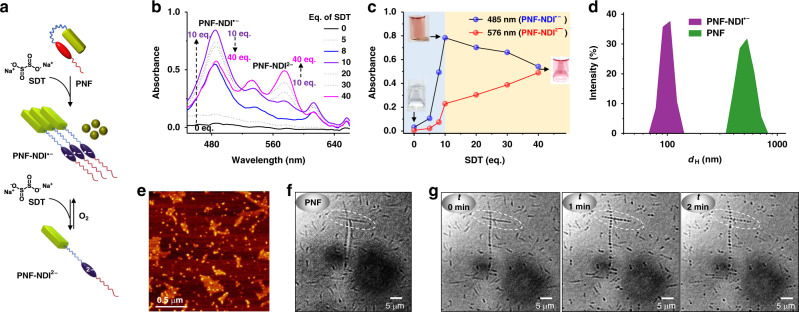
Redox mediated generation of transient-dormant monomer states. **a** Schematic representation of unfolding of PNF CT foldamer upon reduction with SDT to its PNF-NDI^•−^ and PNF-NDI^2−^ reduced states. **b** Absorption spectral changes on addition of varying eq. of SDT to PNF, generating PNF-NDI^•−^ and PNF-NDI^2−^ and **c** corresponding absorbance plot monitoring at the maxima of both reduced states. Photographs of all NDI states are shown in the inset of **c**. **d** DLS spectra indicates the formation of smaller size aggregates of PNF-NDI^•−^ assembly with hydrodynamic diameter (*d*_H_) = 100 ± 25 nm (PDI obtained from DLS (PDI_DLS_) = 0.45) compared to PNF tape structures with *d*_H_ = 554 ± 150 nm (PDI_DLS_ = 0.59), on addition of SDT. **e** AFM image of the unfolded PNF-NDI^•−^ assemblies exhibiting the spherical aggregate morphology. **f**, **g** corresponds to the snapshots, before addition of SDT and after addition of 40 eq. SDT at different time intervals respectively, taken from the Supplementary movie 1. Dotted circle points the surface bound micrometer long tape structures which undergoes disassembly and forms aligned spherical aggregates of PNF-NDI^•−^. The difference in kinetics compared to solution state kinetics is due to different experimental conditions, as for microscopy the solution is more exposed to air and the supramolecular polymers captured are sticking on the surface thus SDT needs to diffuse to the fibers to induce disassembly. [PNF] = 5 × 10^−5^ M, CH_3_CN/H_2_O (1:1 v/v). pH = 8, buffer.

In order to attain the dormant state of PNF, we have exploited the redox behavior of NDI chromophores. NDI chromophores, due to its electron deficient π-core, can be reduced in a sequential and reversible manner to NDI radical anion (NDI^•−^) and dianion (NDI^2−^) states^54^. We envisage that the reduction of the NDI core would simultaneously result in the unfolding of the PNF foldameric amphiphile, due to the disruption of intramolecular CT interactions, as shown in Figs. 1 and  3a. Reduction of PNF (5 × 10^−5^ M), CH_3_CN/H_2_O (1:1 v/v) with sodium dithionite (SDT) was monitored with characteristic spectral features of NDI redox states. With increasing equivalents of SDT, absorption spectra showed a gradual change of PNF-NDI^•−^ absorbance at 485 nm with an initial increase up to 10 eq. of SDT followed by a decrease up to 40 eq. of SDT, whereas the PNF-NDI^2−^ band at 576 nm exhibited a steady hyperchromic shift up to 40 eq. of SDT (Fig. 3b). These changes are accompanied by the disappearance of neutral NDI absorption at 380 nm, depicting the gradual conversion of NDI to NDI^•−^ and subsequently to NDI^2−^ species (See Supplementary Fig. 13c, d). Further, the variation in the composition of different redox states of PNF in solution is also evident from the corresponding color changes as shown in the photographs of solutions at different stages of reduction process (inset, Fig. 3c). The generated PNF-NDI^2−^ species stays in equilibrium with PNF-NDI^•−^ in presence of air and gradually converts to PNF-NDI^•−^ and PNF because of oxidation via slow atmospheric oxygen influx. Hence without oxygen exposure at higher eq. of SDT a stable PNF-NDI^•−^ state was obtained (See Supplementary Fig. 14). PNF-NDI^•−^ absorption maximum showed a gradual red-shift from 482 to 486 nm upon its formation during the titration experiments, with increasing SDT equivalents suggesting the aggregation of unfolded PNF chains in aqueous solution, due to its amphiphilic nature (Fig. 3b). A reversible blue-shift of PNF-NDI^•−^ absorption is observed during the oxidation process from PNF-NDI^•−^ to PNF, reiterating the aggregation of radical anion state (See Supplementary Fig. 15b). The monomeric absorption of PNF-NDI^•−^ at 482 nm is confirmed by the reduction of PNF in CTAB micelles to stabilize the monomeric state^55^, which hardly showed any shift during the reduction-oxidation cycle (See Supplementary Fig. 15). The aggregation of unfolded PNF-NDI^•−^ molecules were also evident from the DLS measurements which showed an average hydrodynamic size of 100 nm upon reduction induced unfolding (Fig. 3d). Further AFM imaging of the unfolded PNF-NDI^•−^ prepared by the drop-casting of corresponding solution followed by immediate drying showed the presence of spherical aggregates, suggesting a different assembly compared to the folded chains (Fig. 3e).

Further, a real time visualization of the reduction induced disassembly process of the 1-D tapes to spherical aggregates upon addition of SDT was attempted through the bright field microscopy. To this end, 40 eq. of SDT was injected to a PNF solution in a closed glass bottom petri dish and time-lapse images were acquired using bright field microscopy. The snapshots from the real time movie at different time points clearly illustrate the reduction induced disassembly of PNF tapes and the subsequent formation of self-assembled, spherical nanoparticles of aggregated PNF-NDI^**• –**^ unfolded molecules (Fig. 3f, g and See Supplementary movie 1, movie 2). The in situ formed spherical particles are appeared to be aligned due to the adhesion of tapes on the glass substrate. These images further suggest that the reduction induced conformational and resultant morphology transformations of PNF foldamer may be an intra-assembly process rather than through unfolded PNF-NDI^**• –**^ monomers. However, the unfolded radical dianion (PNF-NDI^**2 –**^) molecules may exist as monomers due to electrostatic repulsion, which on oxidation would grow on PNF-NDI^**• –**^ particles.

### Transient, Redox-active dormant state of PNF and its kinetically controlled growth

**Fig. 4:**
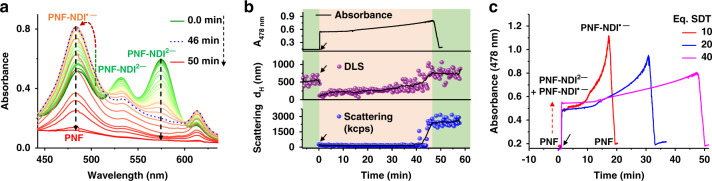
Temporally controlled supramolecular polymerization. **a** Absorption spectral changes during the temporal oxidation of the reduced PNF with 40 eq. SDT. **b** Comparison of time evolution absorbance at 40 eq. SDT with corresponding DLS intensity percentage size data over time along with the scattering (kcps) changes. **c** Time dependent changes in the absorbance of PNF-NDI^•−^ of reduced PNF at 478 nm having 10 eq., 20 eq., and 40 eq. of SDT. Black arrow indicates the point of SDT injection. [PNF] = 5 × 10^−5^ M, CH_3_CN/H_2_O (1:1 v/v), pH = 8, buffer.

Oxidation of PNF-NDI^•−^ in presence of air showed a complete reversible conformational change to the active folded state of PNF as evident from the spectral properties and its self-assembled structures (See Supplementary Fig. 16). Reduced state of unfolded amphiphiles having PNF-NDI^•−^ or PNF-NDI^2−^ states were further explored as monomer reservoirs for the controlled release of active folded conformation of PNF monomers by an oxidation process, to facilitate its kinetically controlled growth. To this end, unfolded dormant states of PNF were generated by the reduction with various eq. of SDT and subsequent oxidation by a slow atmospheric oxygen influx into the solution in an open cuvette (at a constant speed of stirring) to reversibly generate the PNF active monomers with controlled kinetics. The time dependent absorption spectra of the unfolded PNF molecules with 40 eq. SDT (Fig. 4a and See Supplementary Fig. 17) shows firstly an instantaneous formation of the PNF-NDI^•−^ and PNF-NDI^2−^ states as evident from characteristic absorption bands at 485 and 576 nm, respectively, which on oxidation results in gradual conversion of PNF-NDI^2−^ state to PNF-NDI^•−^ state. The differential oxidation rates of PNF-NDI^•−^ and PNF-NDI^2−^ leads to the population of PNF-NDI^**•−**^ state compared to the PNF-NDI^2−^ during the oxidation process. We envisage that this selective stabilization of PNF-NDI^•−^ is due to its aggregation^56^ (vide supra), that protects it from oxidation process whereas, PNF-NDI^2−^ which stays in its monomeric form due to electrostatic repulsion is prone to faster oxidation. On further oxidation, the neutral NDI state is attained in a temporal manner yielding a slow and buffered release of activated PNF monomers that undergo spontaneous supramolecular polymerization (Fig. 4b). To understand more into the self-assembly properties of PNF during the reduction-oxidation process, we have carried out time dependent DLS measurement. Comparison of intensity percentage light scattering size data and the corresponding changes in scattering intensity (kcps) with the time evolution absorption kinetics of PNF during the redox process suggest a good correlation with respect to growth of the supramolecular structures (Fig. 4b). On addition of 40 eq. SDT, there is a sudden decrease in DLS size from average 554 ± 150 nm (PDI_DLS_ = 0.59) to 100 ± 25 nm (PDI_DLS_ = 0.45) and gradually increases on controlled exposure to O_2_ (See Supplementary Fig. 18). During the final stage of the oxidation process DLS size changes occurs with a sigmoidal growth kinetics suggesting a cooperative growth process. In addition, kcps scattering intensity changes over time during the oxidation process follows the similar trend reiterating a controlled growth process on slow oxidation. More importantly, the formation of dormant state, its kinetic stability and its activation to form folded PNF monomers is determined by the amount of SDT and dissolved oxygen. Since, initial amount of dissolved oxygen should not differ significantly, higher eq. of SDT results in consumption of higher oxygen amount at *t* = 0 and also lengthens the existence of PNF-NDI^•−^ and PNF-NDI^2−^ dormant states by their in situ regeneration by the consumption of the excess SDT still present in the solution. As a consequence, a slower kinetics of formation of active PNF molecule and its supramolecular polymerization is obtained. Hence a temporal control over generation of active monomers is achieved via addition of various eq. of SDT (Fig. 4c and See Supplementary Fig. 18).

### Microscopic visualization of the kinetically controlled growth

**Fig. 5:**
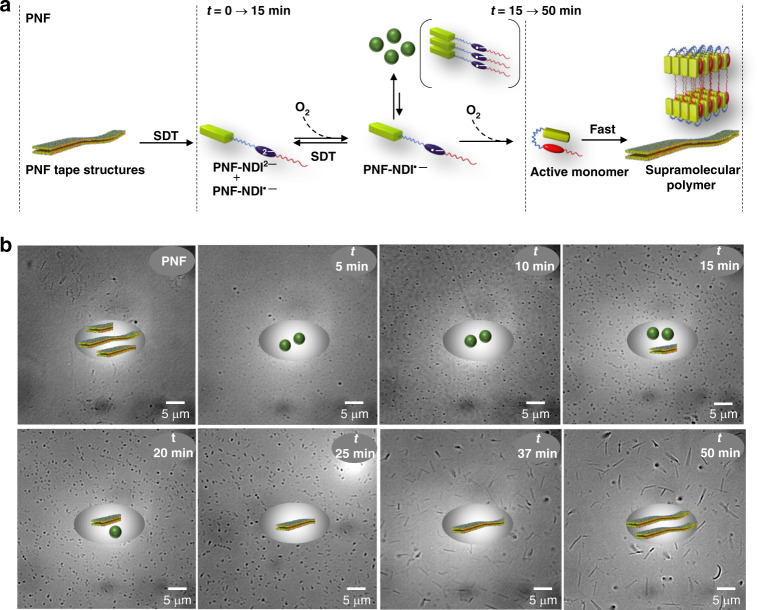
Microscopic visualization of the kinetically controlled growth. **a** Schematic representation of various hierarchical changes during the redox mediated kinetically controlled growth of PNF supramolecular polymers. **b** Bright field microscopic images captured at different time intervals depicting the evolution of the PNF tape structures from the spherical aggregates of PNF-NDI^•−^ assembly. Inset of each image shows the respective time point at which the images were captured along with the schematic of the corresponding structures present in the solution. [PNF] = 5 × 10^−5^ M, CH_3_CN/H_2_O (1:1 v/v), pH = 8, buffer.

We have attempted to visualize the kinetically controlled growth process and its structural implications using bright field microscopy and SIM. In order to avoid surface confinement effects as observed during the redox mediated disassembly (vide supra), controlled growth of the assembly was performed in bulk solution. Aliquots from the bulk solution at different time points during the temporally controlled oxidation aided by the addition of SDT were drop casted on a glass slide for visualization. The total redox sequence is shown with a schematic representation and the corresponding changes in structures is presented by the bright field and SIM images captured at different time scale (Fig. 5a, b and See Supplementary Fig. 19). The bright field image captured after immediate addition of 40 eq. of SDT at *t* = 0 shows the appearance of spherical particles corresponding to the PNF-NDI^•−^ assembly (as seen in AFM earlier, Fig. 3e) which on subsequent oxidation grows bigger in size due to the gradual conversion of PNF-NDI^2−^ to PNF-NDI^•−^ as evident from the image taken at *t* = 5 min. The formation of spherical aggregates could not be visualized through SIM as the Nile red dye emission is quenched during the reduction process.

Further oxidation led to an anisotropic size changes of the particles which is associated with the growth of the folded PNF structures (*t* = 15 min). Accordingly, the appearance of Nile red fluorescence was first observed through SIM mode at this time frame due to the encapsulation of Nile red into the nucleated folded PNF bilayer. Subsequent oxidation processes (*t* = 15 min → 50 min) accompanied with the formation of elongated tape structures with concomitant disappearance of the spherical particles as evidenced from both bright field and SIM microscopic images recorded at different time intervals. Average size of the structures measured from the statistical analysis of the bright field images at different time points showed a reasonably well correlation with the kinetics of growth monitored with absorption and DLS (See Supplementary Fig. 20).

### Control over dispersity and degree of polymerization

Since the assembly of PNF foldamers into 1-D structures is very fast, controlled generation of PNF active monomers via slow oxidation, provides an efficient way to kinetically control the assembly process. Impact of temporally controlled formation and consequent growth of PNF active monomers on the structure of resulting supramolecular assemblies were further investigated using detailed morphological analyses. To rule out the effect of salt concentration arising from the presence of SDT on the structure of assemblies, salt concentration has been maintained constant in all the measurements (protocol for sample preparation is discussed in the “Method” section). TEM studies of the PNF nanostructures formed under kinetic control showed well-defined nanotapes, hinting towards a better structural control (See Supplementary Fig. 21).

**Fig. 6:**
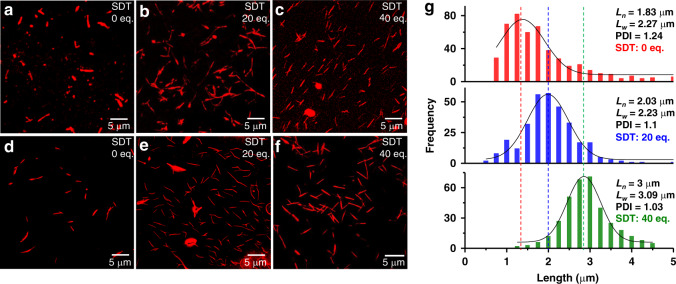
Structural parameters of PNF supramolecular polymers. Confocal laser scanning microscopy (CLSM) images of **a** instantaneously prepared PNF assemblies with 0 eq. SDT, **b** kinetically grown tape structures using 20 eq. of SDT and **c** kinetically grown structures with 40 eq. SDT. **d**–**f** are corresponding structured illumination microscopic (SIM) images. **g** Histogram showing the length distribution of the tapes for instantaneously grown structures from top to bottom at 0 eq. SDT (PDI = 1.24, *L*_w_ = 2.27 µm, *L*_n_ = 1.83 µm, *n* = 473, red color histogram), kinetically controlled growth of tapes at 20 eq. SDT (PDI = 1.1, *L*_w_ = 2.23 µm, *L*_n_ = 2.03 µm, *n* = 472, blue color histogram) and at 40 eq. SDT (PDI = 1.03, *L*_w_ = 3.09 µm, *L*_n_ = 3 µm, *n* = 350, green color histogram), obtained from the SIM images. The dotted lines are drawn from the maximum for each eq. of PDI distribution to guide the eye. *L*_w_ = weight-average length, *L*_n_ = number-average length, *n* = number of tapes counted, PDI = polydispersity index. [PNF] = 5 × 10^−5^ M, CH_3_CN/H_2_O (1:1 v/v), pH = 8, buffer. Fluorescent probe: 5 µM Nile red, *λ*_exc_ = 561 nm.

In an attempt to acquire quantitative information on the degree of polymerization and dispersity of the kinetically formed PNF supramolecular polymers, we have visualized Nile red loaded structures in solution using confocal laser scanning microscopy (CLSM) (Fig. 6a–c). Images indeed showed a uniform distribution of the dye and the presence of monodisperse 1-D structures when formed under kinetically controlled oxidation conditions in agreement with the light-scattering studies. In order to perform accurate statistical analysis on the structure, we have performed SIM with better resolution (~120 nm, Fig. 6d–f). A frequency statistics on the length of supramolecular polymers was performed on the resulting images and their number (*L*_n_) and weighted average (*L*_w_) were calculated and the corresponding PDI was determined as *L*_w_/*L*_n_. The tape structures formed by instantaneous preparation show polydisperse structures having a wide distribution of lengths ranging from 0.5 to 6 µm (Fig. 6a, d and See Supplementary Fig. 22). The number-average length (*L*_n_) and weight-average length (*L*_w_) of these structures were calculated to be 1.83 and 2.27 μm, respectively with a PDI of 1.24 (Fig. 6g). On the other hand, the kinetically grown tape structures show a gradual increase in the average degree of polymerization with increase in the equivalent of SDT, as evident from their *L*_n_ values which increase to 2.03 and 3 μm, with 20 and 40 equivalents of SDT (Fig. 6g, See Supplementary Figs. 23 and 24, and See Supplementary Table 1). In the presence of high concentration of SDT, a slower oxidation rate is observed that ensures a slower release of active monomers, as a result of which they tend to grow on the existing chains rather than initiating the growth of another chain. This clearly suggests the importance of a kinetically controlled polymerization for achieving higher degree of polymerization compared to the random assembly process.

Remarkably, PDIs of the corresponding tape structures were calculated to be 1.1 and 1.03, suggesting highly monodisperse structures. This decrease in PDI for kinetically grown structures validates our approach of growing the tape structures under kinetic control using the redox-responsive dormant states. Interestingly, the redox process is reversible and could be repeated multiple times (See Supplementary Fig. 18c) depicting no effect of redox agents on the system.

## Discussion

In conclusion, we present the temporal supramolecular polymerization of an amphiphilic charge-transfer monomer (PNF) utilizing a unique transient, redox-responsive dormant state. The transient-dormant state for the CT monomers was generated by reducing the acceptor component NDI with SDT to produce the corresponding radical anion species which under deoxygenated conditions show sufficient stability owing to its aggregation. This inactive redox-dormant state of PNF upon exposure to atmospheric oxygen sequesters active monomer molecules that undergo kinetically controlled supramolecular polymerization resulting in highly monodisperse CT supramolecular polymers spanning in micrometer length scale. Further we have shown that the degree of polymerization and dispersity of these assemblies could be modulated with the rate of oxidation using various concentration of the reducing agent. Our approach demonstrates the use of redox chemistry in controlling the growth of supramolecular polymers, which can be generalized to any electronically active monomers irrespective of its structure. The redox-responsive dormant states of assembled radical anions presented here are indeed out-of-equilibrium transient states and hence are analogous to the metastable dormant states formed under kinetic conditions for various chromophoric systems. Hence this approach suggest the use of transient assemblies^57–59^ as an alternative way of achieving high-energy dormant states for monomers of highly dynamic supramolecular polymers, where metastable dormant states cannot be achieved under kinetic conditions. Further, this approach can be extended to multicomponent assemblies with predictive sequence using monomers with different redox activities and also to nonequilibrium assemblies by coupling to redox cycles^43^.

## Methods

### Procedure of sample preparation for instantaneously prepared tape structures

The required amount of PNF for 5 × 10^−5^ M concentration was taken in pure acetonitrile. On the other hand, to an aqueous buffer solution (pH = 8) required amount of SDT was added and oxidized completely by shaking in exposure to air and waited for sufficient time. This stock of aqueous buffer solution containing required amount of oxidized SDT salt was used to make the final solvent composition CH_3_CN/H_2_O (1:1 v/v), for PNF to perform the studies.

### Procedure for instantaneous preparation of tape structures with Nile red dye as fluorescent probe

The 100 µL of required amount of PNF for 5 × 10^−5^ M concentration in spectroscopic grade acetonitrile was mixed with 5 µM Nile red dye in 25 µL of THF and shaken properly. The solution mixture was evaporated and dried in vacuum to get rid of THF solvent. Finally, the dried film was redissolved in required amount of acetonitrile and mildly sonicated. To the final solution water (buffer solution, pH = 8) containing eq. amount of oxidized SDT salt was added to produce Nile red loaded tape structures.

### Procedure for preparations of kinetically grown tape structures with Nile red dye as fluorescent probe (40 eq. of SDT)

The 100 µL of required amount of PNF for 5 × 10^−5^ M concentration in spectroscopic grade acetonitrile was mixed with 5 µM Nile red dye in 25 µL of THF and shaken properly. The solution mixture was evaporated and dried in vacuum to get rid of THF solvent. Finally, the dried film was redissolved in required amount of acetonitrile and mildly sonicated followed by addition of aqueous buffer solution. Then freshly prepared 20 µL solution of SDT (40 eq.) made in aqueous buffer solution (stock concentration = 220 mM, pH = 8) was added and studied for temporal oxidation. Similar procedure was followed for kinetically grown tape structures with other eq. of SDT as well.

## Supplementary information

Supplementary Information

Description of Additional Supplementary Files

Supplementary Movie 1

Supplementary Movie 2

## Data Availability

All data supporting the findings are available in the article as well as the Supplementary Information files from the authors on reasonable request.
