# Supplementary material for: Transient dormant monomer states for supramolecular polymers with low dispersity
Source: Nat Commun. 2020 Aug 7;11:3967. doi: 10.1038/s41467-020-17799-w (PMC7415150; doi:10.1038/s41467-020-17799-w)
Supplement: Supplementary file 1 — Supplementary Information [file 41467_2020_17799_MOESM1_ESM.pdf]

Supplementary Information

Transient Dormant Monomer States for Supramolecular Polymers with Low Dispersity

Jalani *et al.*

Supplementary Methods

NMR measurements: NMR spectra were recorded with a Bruker AVANCE 400 (400 MHz) Fourier transform NMR spectrometer with chemical shifts reported in parts per million (ppm) with respect to TMS. Splitting patterns are designated as s, singlet; d, doublet; bs, broad singlet; m, multiplet; t, triplet.

High Resolution Mass Spectrometry (HRMS): High Resolution Mass Spectra (HRMS) were recorded on an Agilent 6538 Ultra High Definition (UHD) Accurate-Mass Q-TOF-LCMS system using electrospray ionization (ESI) technique either in positive mode or negative mode.

Spectroscopic measurements: UV-Visible absorption spectra were recorded on a Perkin Elmer Lambda 900 UV-Vis-NIR Spectrometer and emission spectra were recorded on Perkin Elmer LS 55 Luminescence Spectrometer. Fluorescence spectra of solutions were recorded with 320 nm excitation wavelength. 10 mm x 10 mm quartz cuvettes were used for UV-vis and fluorescence measurements.

Transmission Electron Microscopy (TEM) measurements: Transmission Electron Microscopy (TEM) measurements were performed on JEOL JEM 3010 operated at 300 kV. Samples were prepared by placing a drop of solution on carbon coated copper grids followed by drying at room temperature. The images were recorded with an operating voltage of 300 kV. In order to get a better contrast, the samples were stained with uranyl acetate (0.1 wt % in water) before the measurements.

Atomic Force Microscopy (AFM): Atomic Force Microscopy (AFM) measurements were performed on a Veeco di Innova SPM operating in tapping mode regime. Micro-fabricated silicon cantilever tips doped with phosphorus with frequency between 235 and 278 kHz and a spring constant of 20-40 Nm^{-1} were used. The samples for the tape structures were prepared by drop casting the solution on silicon substrate and dried in air followed by vacuum drying at room temperature. On the other hand, AFM images for the spherical aggregates of **PNF** radical anion assembly was recorded by dropcasting 15 μL of the freshly reduced solution onto the mica substrate and immediately soaking out the excess sample with tissue paper followed by drying with N_2 gas blowing.

Dynamic light scattering measurements: Dynamic Light Scattering (DLS) measurements were carried out using a NanoZS (Malvern UK) employing a 633 nm laser at a back scattering angle of 173°.

Confocal microscopy: Confocal microscopy imaging was done at room temperature using a Zeiss LSM 510 META laser scanning confocal microscope with a laser excitation of λ_{exc} = 561 nm and emission

collection in the range of 570 nm to 650 nm for Nile red dye. The microscope objective of 63X (NA 1.4) and 100X (NA 0.5) were employed. The images were captured by drop casting the freshly prepared sample on a glass bottom petridish and closed with cap to avoid drying.

Structured Illumination Microscopy (SIM) measurements: Structured Illumination Microscopy (SIM) images were captured using an inverted Zeiss ELYRA PS1 microscope. The prepared sample solution was dispersed on a coverslip attached with a 35 mm dish and kept under the microscope. Fluorescence microscopic images were captured by structured illumination method using laser excitation at λ_{exc} = 561 nm (200 mW) and emission collection in the range of 570 nm to 650 nm for Nile red dye. Imaging was performed using 10% of the incident laser (~ 0.5 kW.Cm^{-2}) using Zeiss oil–immersion objective (alpha Plan–apochromat DIC 63x/1.40 Oil DIC M27, numerical aperture (NA) 1.40 oil). Fluorescence light was spectrally filtered with emission filters (MBS– 561+EF BP 570–650/LP 750 for laser line 561 and imaged using a PCO edge sCMOS camera. The images were captured by drop casting the freshly prepared samples on glass bottom petridish and closed with cap to avoid drying and O_2 exposure. For time dependent monitoring of growth process of the fluorescent tape structures, the images were captured after drop casting the 15 μL of sample onto the glass slide which was immediately covered with the glass cover slip avoiding fast drying and exposure to air.

Synthesis: Synthesis of **PNF** and **Pyrene-HEG** was performed according to the Supplementary Figures 1-4 and corresponding procedures and characterizations are given below each Figure.

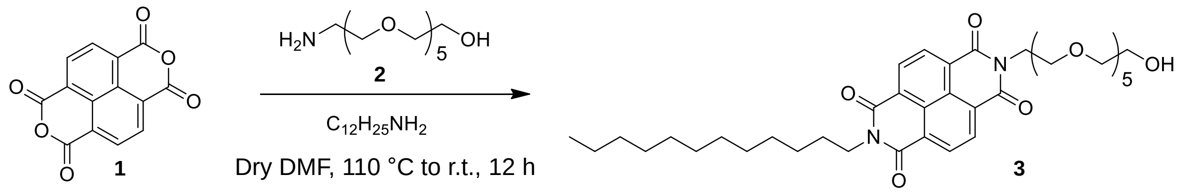

Supplementary Figure 1. Synthetic route to molecule **3**.

Synthetic procedure of **3**: Dodecylamine (1.38 g, 7.44 mmol), 1,4,5,8-naphthalenetetracarboxylic dianhydride (**1**) (2.0 g, 7.45 mmol) and compound **2** (2.1 g, 7.46 mmol) were mixed together in 70 mL dry DMF. The reaction mixture was refluxed at 110 °C for 12 h under inert atmosphere. The solvent was then evaporated under high vacuum. The resulting residue was dissolved in chloroform and filtered. The filtrate was evaporated and purified by column chromatography (silica, 100-200 mesh) with 5% methanol in chloroform (v/v) solvent mixtures to yield 17 % of the pure product. ^{1}H NMR (400 MHz, $CDCl_3$, TMS) : δ (ppm) 8.75 (s, 4H), 4.44 (t, J = 8 Hz, 2H), 4.19 (t, J = 8 Hz, 2H), 3.85 (t, J = 6 Hz, 2H), 3.69 (t, J = 6.4 Hz, 2H), 3.65-3.57 (m, 20H), 1.78 – 1.70 (m, 2H), 1.46 – 1.25 (m, 18H) 0.87 (t, J = 7.2 Hz, 3H); ^{13}C NMR (100 MHz, $CDCl_3$): δ (ppm) 162.91, 162.81, 130.98, 130.91, 126.76, 126.68, 126.55, 72.68, 70.54, 70.42, 70.16, 70.02, 67.81, 61.65, 41.01, 39.55, 31.89, 29.60, 29.51, 29.32, 27.07, 22.66, 14.10. HRMS: m/z calculated: $C_{38}H_{58}N_3O_{10}$:716.4122, found: 716.4116 $[M+NH_4]^+$.

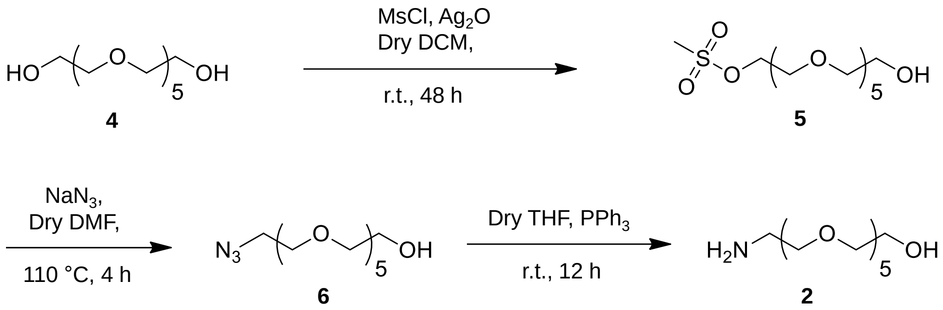

Supplementary Figure 2. Synthetic route to hexaethylene glycol amine (**2**).

Molecule **2** was synthesized following the reported procedure.[1]

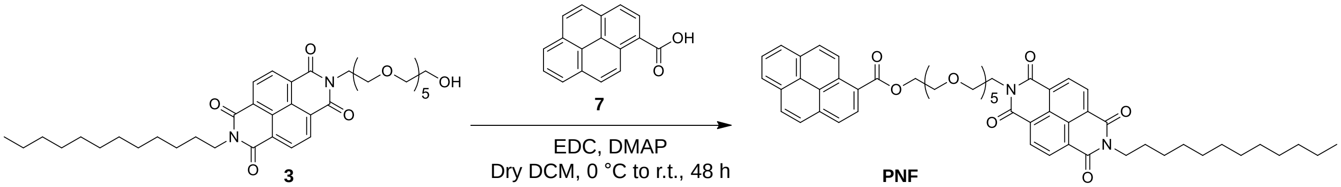

Supplementary Figure 3. Synthetic route to molecule **PNF**.

Synthetic procedure of **PNF:** Molecule **3** (425.6 mg, 0.61 mmol) and **7** (100 mg, 0.41 mmol) were taken in 15 mL ice cold dry dichloromethane and stirred for 15 min. In another set up, EDC (N-(3-Dimethylaminopropyl)-N′-ethylcarbodiimide hydrochloride) (100 mg, 0.52 mmol) and 35 mg (0.28 mmol) of DMAP (4-Dimethylaminopyridine) were taken in 15 mL of dry dichloromethane and added to the initial mixture. This reaction mixture was kept in ice for 15 min and then allowed to stir at room temperature for 48 h. The reaction mixture was then extracted with dichloromethane and water (30 mL x 3). The organic layer was dried under vacuum and purified by column chromatography (silica gel, 100-200 mesh) with 2 % methanol in chloroform to give 220 mg of the pure product (yield: 60%). ^{1}H NMR (400 MHz, $CDCl_3$, TMS) : δ (ppm) 8.97 (d, J = 9.6 Hz, 1H), 8.49 (d, J = 8 Hz, 2H), 8.26 (s, 4H), 8.09 (d, J = 7.2 Hz, 1H), 8.08 (d, J = 7.6 Hz, 1H), 7.98-7.94 (m, 4H), 4.67 (t, J = 4.8 Hz, 2H), 4.37 (t, J = 5.6 Hz, 2H), 4.08 (t, J = 7.6 Hz, 2H), 3.84 (t, J = 4 Hz, 2H), 3.84 – 3.55 (m, 18H), 1.78 – 1.70 (m, 2H), 1.43 – 1.25 (m, 18H), 0.87 (t, J = 7.2 Hz, 3H); ^{13}C NMR (100 MHz, $CDCl_3$): δ (ppm) 167.45, 162.5, 162.41, 133.82, 130.60, 130.56, 130.1, 130.01, 129.93, 129.41, 129.08, 128.45, 126.92, 126.3, 126.21, 126.09, 125.69, 125.62, 125.58, 70.83, 70.76, 70.73, 70.70, 70.68, 70.60, 70.57, 70.18, 69.35, 67.85, 64.36, 40.85, 39.52, 31.89, 29.62, 29.6, 29.58, 29.54, 29.31, 28.06, 27.14, 22.64, 14.06. HRMS: m/z calculated: $C_{55}H_{66}N_3O_{11}$: 944.4697, found: 944.4694 $[M+NH_4]^+$.

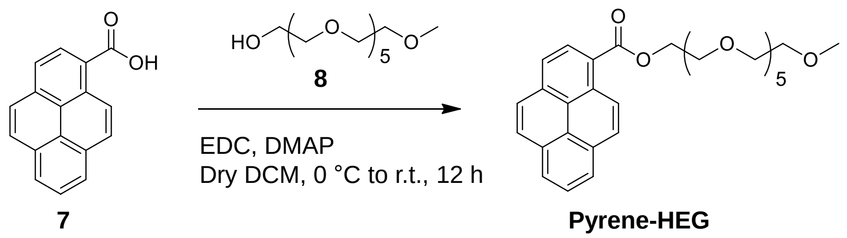

Supplementary Figure 4. Synthetic route for the molecule **Pyrene-HEG**.

Synthetic procedure of **Pyrene-HEG**: Monomethylated derivative of hexaethylene glycol (**8**), (310 mg, 1.04 mmol) and compound **7** (172 mg, 0.7 mmol) were taken in 15 mL ice-cold dry dichloromethane and stirred for 15 min. In another set up, EDC (N-(3-Dimethylaminopropyl)-N'-ethylcarbodiimide hydrochloride) (168 mg, 0.87 mmol) and DMAP (4-Dimethylaminopyridine) (60 mg, 0.49 mmol) were taken in 15 mL of dry dichloromethane and added to the initial mixture. This reaction mixture was kept in ice for 15 min and then allowed to stir at room temperature for 12 h. The reaction mixture was then extracted with dichloromethane and water (30 mL x 3). The organic layer was then dried under vacuum and purified by column chromatography (silica gel, 100-200 mesh) with 5 % methanol in chloroform to give 160 mg of the pure product (yield: 45 %). ^{1}H NMR (400 MHz, $CDCl_3$, TMS): δ (ppm), 9.27 (d, J = 9.2 Hz, 1H), 8.66 (d, J = 8 Hz, 1H), 8.28 – 8.23 (m, 3H), 8.17 (m, 2H), 8.11 – 8.04 (m, 2H), 4.66 (t, J = 8 Hz, 2H), 3.96 (t, J = 5.6 Hz, 2H), 3.78-3.50 (m, 20H), 3.35 (s, 3H); ^{13}C NMR (100 MHz, $CDCl_3$): δ (ppm), 167.95, 134.34, 131.14, 130.99, 130.37, 129.62, 129.43, 128.55, 127.17, 126.29, 126.27, 126.16, 124.93, 124.81, 124.18, 124.11, 123.50, 71.87, 70.72, 70.65, 70.50, 70.45, 69.31, 64.28, 58.99. HRMS: m/z calculated: $C_{30}H_{40}NO_8$: 542.2754, found: 542.2750 $[M+NH_4]^+$

Packing factor calculation for folded **PNF**. The packing factor (f) [2-6] was calculated using the following Supplementary Equation 1.

$$f = \frac{\boldsymbol{v}}{\boldsymbol{al}} \quad (1)$$

$$v = 27.4 + 26.9\,n \quad (2)$$

$$l = 1.5 + 1.265\,n \quad (3)$$

Here $\boldsymbol{l}$ corresponds to the hydrophobic chain length of the molecule and $\boldsymbol{a}$ is the ratio of hydrophilic to hydrophobic interfacial area. For an amphiphile with hydrophobic alkyl chain, the hydrophobic chain length ($\boldsymbol{l}$) and hydrophobic volume ($\boldsymbol{v}$) can be calculated by using the Tanford equations (Supplementary Equation 2 and Supplementary Equation 3)[7] where, $\boldsymbol{n}$ corresponds to the number of carbon atoms in the alkyl chain.

The ratio of hydrophilic to hydrophobic interfacial area ($\boldsymbol{a}$) was calculated as

$$a = \pi\left(\frac{d}{2}\right)^2 \quad (4)$$

where d corresponds to the diameter of the interfacial area.

Here, the total hydrophobic length of **PNF** foldamer includes the C_{12} alkyl chain along with NDI and pyrene components. Thus a total length of $\boldsymbol{l} = 24.27$ Å was measured for hydrophobic segment from the energy minimized structure which corresponds to the hydrophobic chain length equivalent to $n = 18$. Hence, using the Supplementary Equation 2 and Supplementary Equation 3, the hydrophobic volume for C_{18} alkyl chain was calculated to be $\boldsymbol{v} = 511.6$ Å^3 with length $\boldsymbol{l} = 24.27$ Å.

a was calculated as $= \pi\left(\frac{d}{2}\right)^2 = \pi\left(\frac{5.04}{2}\right)^2 = 19.94$ Å^2

d was obtained from the energy minimized structure of **PNF** generated from Chem 3D pro.12 software.

Hence the packing factor (*f*) was calculated to be,

$$f = \frac{\boldsymbol{v}}{\boldsymbol{al}} = \frac{511.6\ \text{Å}^3}{(19.94\ x\ 24.27)\text{Å}^3} = 1.05$$

This value of f = 1.05 correlates well with the lamellar packing of the typical amphiphiles.[6]

Length analysis of SIM microscopic data: Length analysis of SIM images for individual tape structures were done using freeware ImageJ software. A frequency statistics was done for the obtained lengths and their number average (L_n) and weight average (L_w) was calculated according to the following Supplementary Equations 5 and 6.

$$L_n = \frac{\sum_{i=1}^{n} N_i L_i}{\sum_{i=1}^{n} N_i} \quad (5)$$

$$L_w = \frac{\sum_{i=1}^{n} N_i L_i^2}{\sum_{i=1}^{n} N_i L_i} \quad (6)$$

PDI was calculated by L_w/L_n where N_i is the frequency of length L_i.

Supplementary Figures

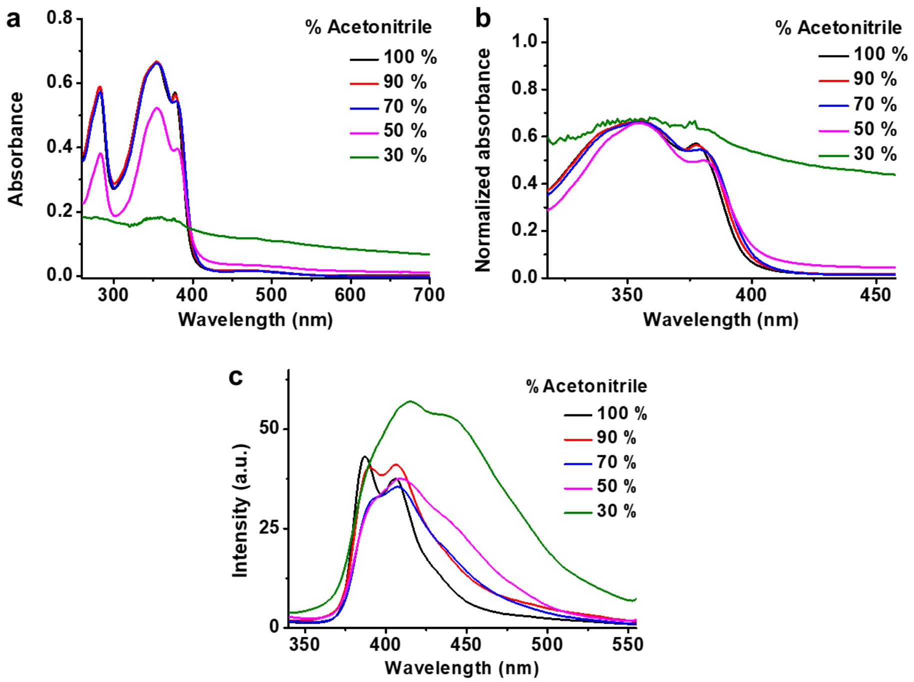

Supplementary Figure 5. Solvent composition dependent absorption and emission spectra of **PNF**. **a.** absorption, **b.** normalized absorption and **c.** corresponding emission spectra (λ_{exc} = 300 nm) of **PNF** at various percentages of CH_3CN in H_2O showing a gradual aggregation with increasing amount of H_2O. [**PNF**] = 5 x 10^{-5} M.

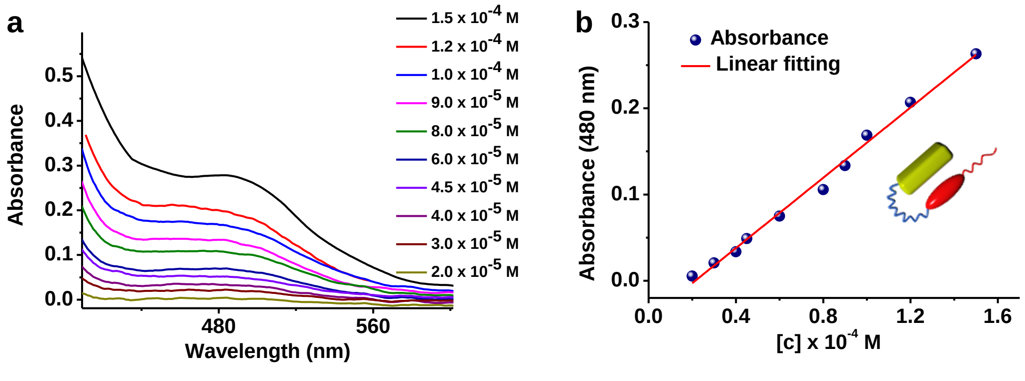

Supplementary Figure 6. a. and **b.** are the concentration dependent absorption spectra of **PNF** in CH_3CN/H_2O (1:1 v/v) showing a linear trend in the CT absorption at 480 nm which suggest an intramolecular CT interaction leading to a foldamer conformation.

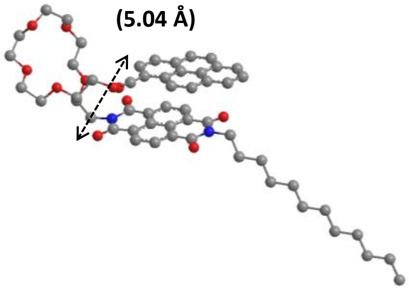

Supplementary Figure 7. Optimized molecular model for the folded **PNF** obtained after energy minimization using Chem 3D pro.12 software. The dashed arrow indicates diameter (*d*) of the hydrophobic-hydrophilic interfacial area.

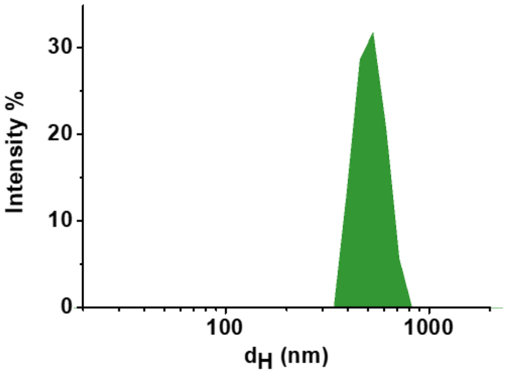

Supplementary Figure 8. Dynamic light scattering (DLS) measurement of the instantaneously formed assembly of **PNF** with d_H = 554 ± 150 nm (PDI_{DLS} = 0.59), in CH_3CN/H_2O (1:1 v/v). [**PNF**] = 5 x 10^{-5} M.

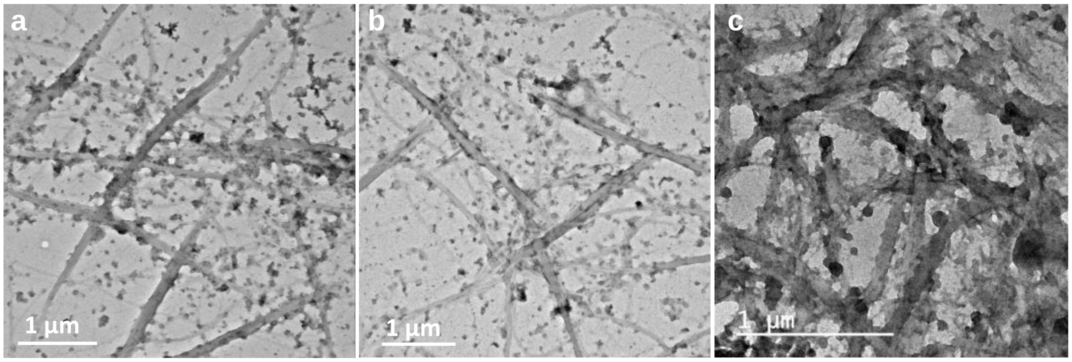

Supplementary Figure 9. a-c. are the TEM images for the instantaneously grown **PNF** tape structures. [**PNF**] = 5 x 10^{-5} M, CH_3CN/H_2O (1:1 v/v).

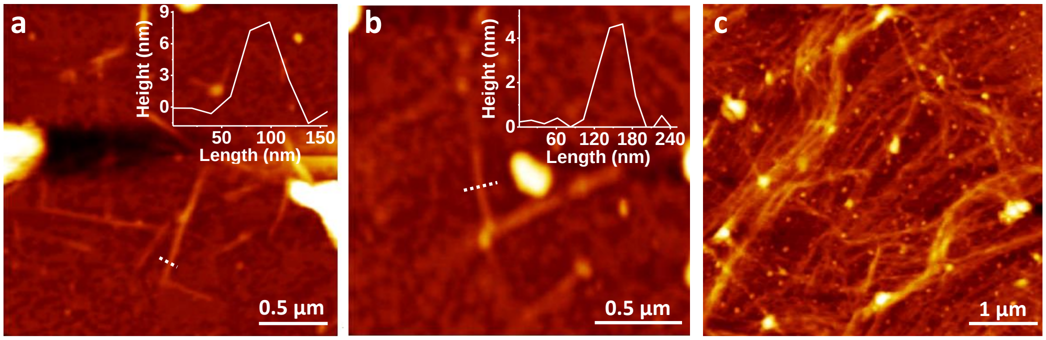

Supplementary Figure 10. **a-c**. AFM height images for the instantaneously prepared tape structures of **PNF**. Inset of **a** and **b** shows the height analyses of the tape structures along the dotted lines shown in the images, suggesting the bilayer packing in these tapes. Silicon substrate was used for drop-casting the sample solution. [**PNF**] = 5 x 10^{-5} M, CH_3CN/H_2O (1:1 v/v).

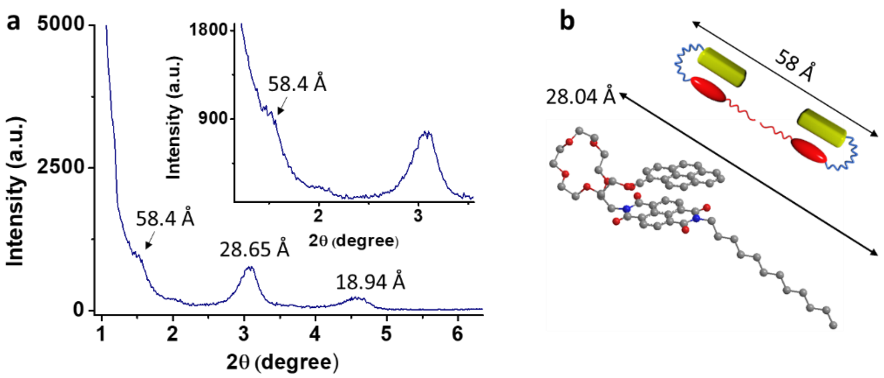

Supplementary Figure 11. Bilayer packing of the foldameric **PNF** amphiphile. **a.** Thin film XRD spectrum for the **PNF** tape structures showing a maximum *d*-spacing of 58.4 Å corresponding to the height of the bilayer packing of **PNF** amphiphiles in tape. Inset shows the zoomed portion showing small angle peaks corresponding to the *d*-spacing of 28.65 Å and 18.94 Å which correlates well with the XRD-pattern for lamellar organization of the bilayers. **b.** Schematic shows the bilayer packing distance of 58 Å, and molecular dimension of 28.04 Å, calculated from the CPK model structures using 3D chem draw software.

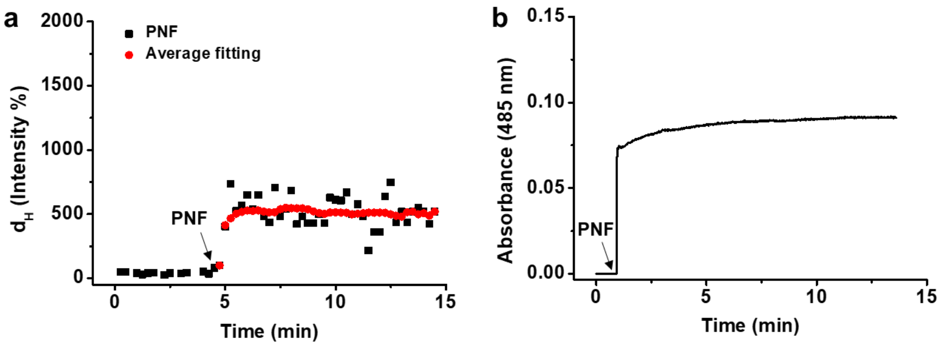

Supplementary Figure 12. Instantaneous assembly of **PNF**. **a.** Time dependent DLS measurement for **PNF** suggests the instantaneous growth of **PNF** CT-assembly in solution and average size remains same throughout the measurement. **b.** Time dependent absorption spectra for **PNF**, monitored at the CT band (485 nm). Arrow indicates the injection of **PNF** stock solution in the solvent mixture. The measurements were done immediately after preparing the sample solution. [**PNF**] = 5 x 10^{-5} M, CH_3CN/H_2O (1:1 v/v).

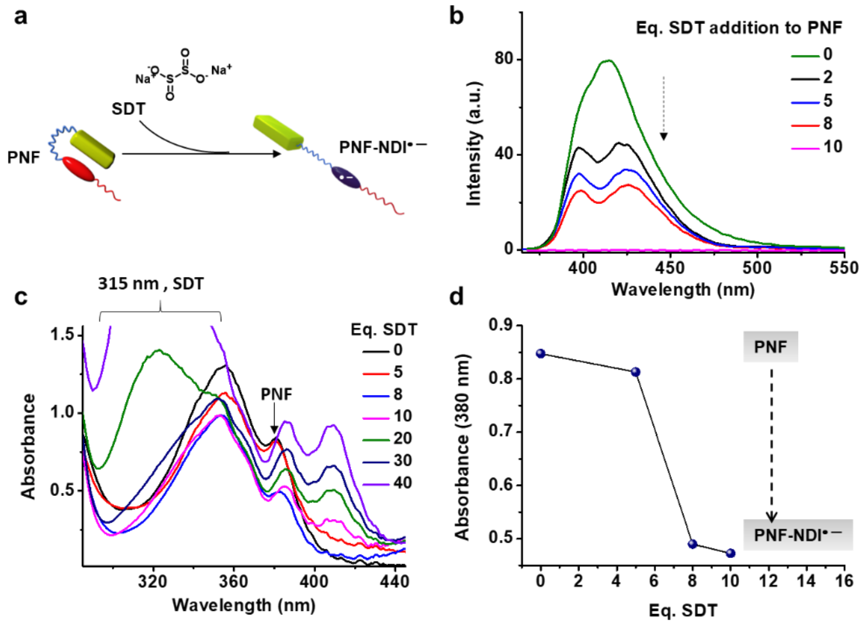

Supplementary Figure 13. a. Schematic representation for the unfolding process after reducing **PNF** with SDT generating **PNF-NDI$^{\bullet-}$**. **b.** Quenching of pyrene emission on addition of SDT to **PNF**. Since the pyrene emission is quenched in the presence of SDT, it could not be used as a probe for unfolding process. **c.** Absorption spectral changes on addition of various eq. of SDT showing the conversion of NDI to **PNF-NDI$^{\bullet-}$** leading to a gradual decrease in NDI absorbance at 380 nm. The band evolved at λ = 315 nm corresponds to SDT absorption and **d.** shows the corresponding absorbance versus eq. of SDT plot monitored at 380 nm corresponding to the NDI chromophores. [**PNF**] = 5 x 10^{-5} M, CH_3CN/H_2O (1:1 v/v).

Supplementary Note 1**:** the source of weak pyrene emission for **PNF** is because of the residual pyrene which is weakly bound with NDI and not involved in CT co-assembly, proved by lifetime measurement.

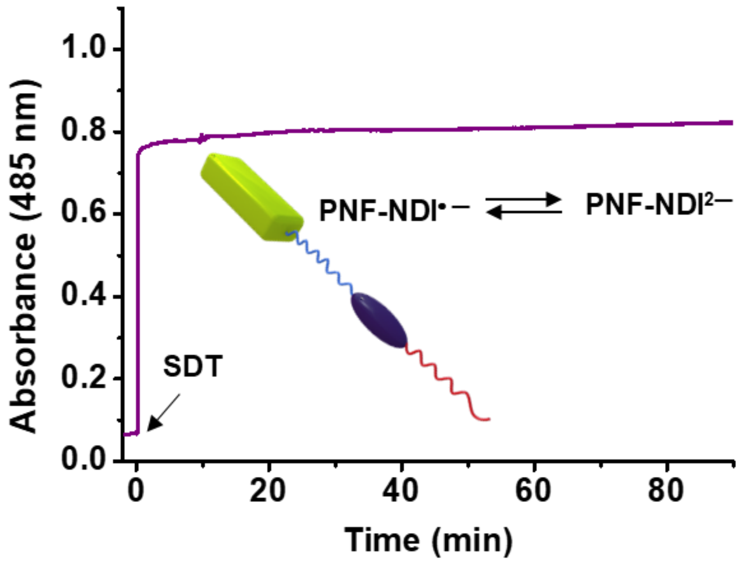

Supplementary Figure 14. Time dependent absorbance changes of **PNF-NDI$^{\bullet-}$** in a closed cuvette without exposure to air, monitored at 485 nm (40 eq. SDT) suggesting its stability in the assembled state. [**PNF**] = 5 x 10^{-5} M, CH_3CN/H_2O (1:1 v/v). pH = 8, buffer.

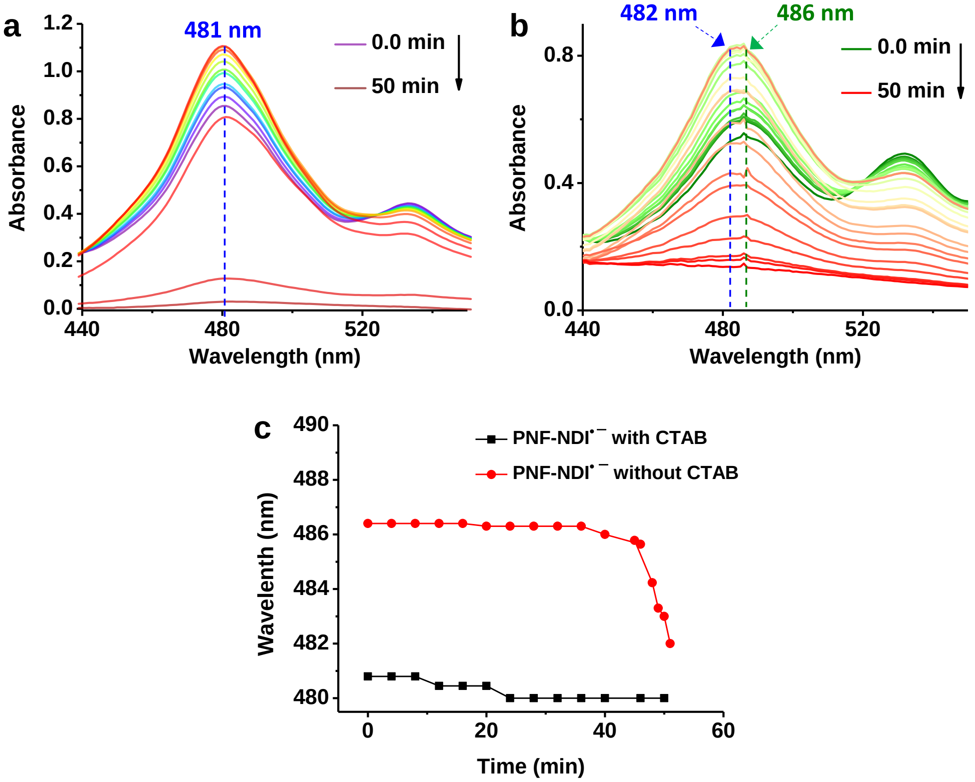

Supplementary Figure 15. Time dependent spectral changes of the **PNF-NDI**$^{\bullet-}$ species upon temporal oxidation by atmospheric oxygen, **a.** in presence of CTAB, **b.** without CTAB. **c.** Plot of **PNF-NDI**$^{\bullet-}$ wavelength maximum against time which shows a blue shift of the **PNF-NDI**$^{\bullet-}$ absorbance during oxidation process suggesting that **PNF-NDI**$^{\bullet-}$ is indeed aggregated at the beginning in absence of CTAB. On the other hand, in presence of CTAB, **PNF-NDI**$^{\bullet-}$ shows no blue shift during oxidation process and exists as monomer. The dotted green line in (**b**) shows the wavelength maximum of **PNF-NDI**$^{\bullet-}$ immediately after reduction whereas the dotted blue line corresponds to the wavelength maximum for the final oxidation process. [**PNF**] = 5 x 10^{-5} M, CH_3CN/H_2O, (1:1, v/v). [SDT] = 40 eq., [CTAB] = 0.05 M.

Supplementary Note 2: In the presence of CTAB micelles, the generated **PNF-NDI**$^{\bullet-}$ assembly dissociates and stays in the micellar pocket as an individual **PNF-NDI**$^{\bullet-}$ giving rise to monomeric absorption.

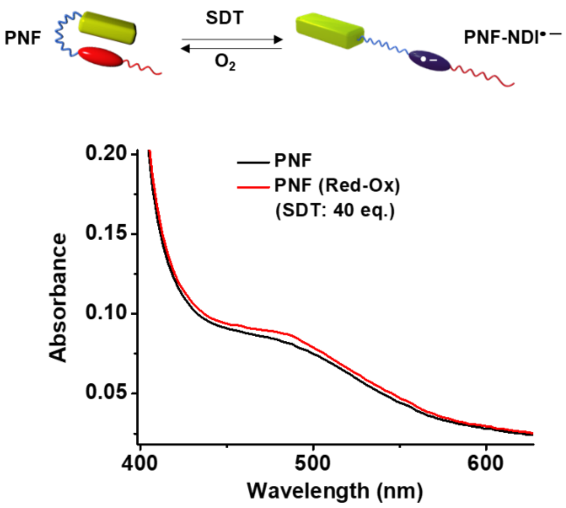

Supplementary Figure 16. Absorption spectra of instantaneously prepared **PNF** and the **PNF** obtained after one reduction-oxidation cycle in solution using 40 eq. SDT showing there is hardly any difference in the extent of CT interactions. [**PNF**] = 5 x 10^{-5} M, CH_3CN/H_2O (1:1 v/v).

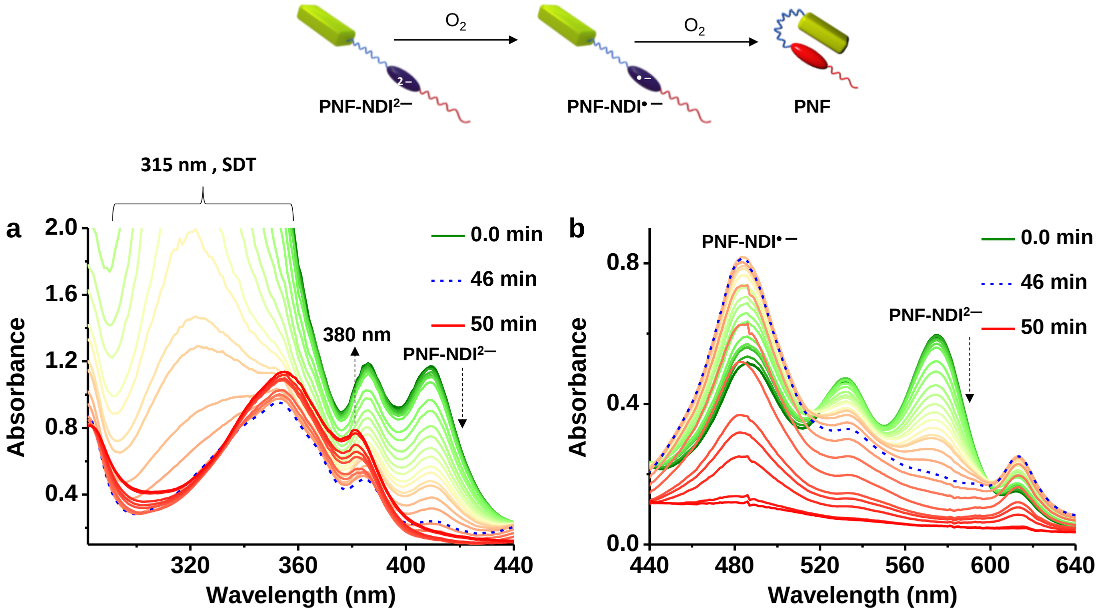

Supplementary Figure 17. a. and **b.** are the time evolution spectral changes of the **PNF-NDI^{2-}** species over temporal oxidation by atmospheric oxygen at different wavelength ranges. The absorption band at 315 nm arises due to SDT. Figure **a.** shows the gradual evolution of neutral NDI (**PNF**) band at 380 nm and disappearance of **PNF-NDI^{2-}** band at 420 nm during the oxidation process. **PNF-NDI^{2-}** was generated with 40 eq. SDT. [**PNF**] = 5 x 10^{-5} M, CH_3CN/H_2O (1:1 v/v).

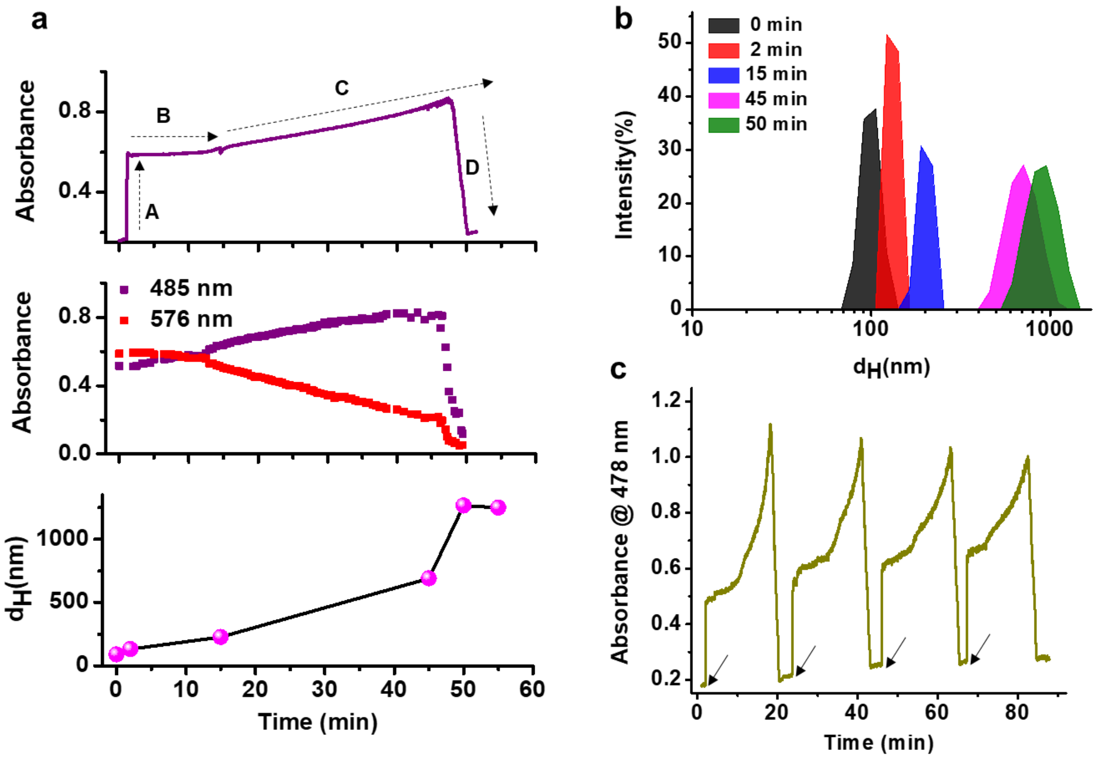

Supplementary Figure 18. Comparison of absorbance and DLS size changes of **PNF** over time during redox process. **a.** Comparison of kinetics (top to bottom) showing, time evolution absorption changes of **PNF** at 40 eq. SDT, monitored at 485 nm, showing various steps of reduction and oxidation process denoted as A, B, C and D guided with arrows (top), time evolution absorption changes of **PNF** at 40 eq. SDT, monitored at both 485 nm (**PNF-NDI**$^{\bullet-}$), 576 nm (**PNF-NDI**$^{2-}$) band (middle) and hydrodynamic radius from DLS measurements (bottom) for **PNF** at 40 eq. SDT monitored at various time intervals during its reduction-oxidation process. **b.** DLS measurements at various stages of reduction and oxidation process (Step A to Step D) corresponding to the DLS data plot in **a** (bottom) against time. t = 0 min, Size: 100 ± 25 nm (PDI_{DLS} = 0.45); t = 2 min, Size: 135 ± 20 nm (PDI_{DLS} = 0.40); t = 15 min, Size: 228 ± 23 nm (PDI_{DLS} = 0.40); t = 45 min, Size: 743.8 ± 216.8 nm (PDI_{DLS} = 0.39); t = 50 min, Size: 1107.7 ± 265 nm (PDI_{DLS} = 0.21). **c**. Multiple redox cycle of **PNF** using 10 eq. of SDT. [**PNF**] = 5 x 10^{-5} M, CH_3CN/H_2O (1:1 v/v). The reversibility of the redox cycle works well below 80 eq. SDT.

Supplementary Note 3: Generation of NDI reduced species from **PNF** with concomitant unfolding of the foldamer and various steps of reduction and oxidation kinetics is explained as follows.

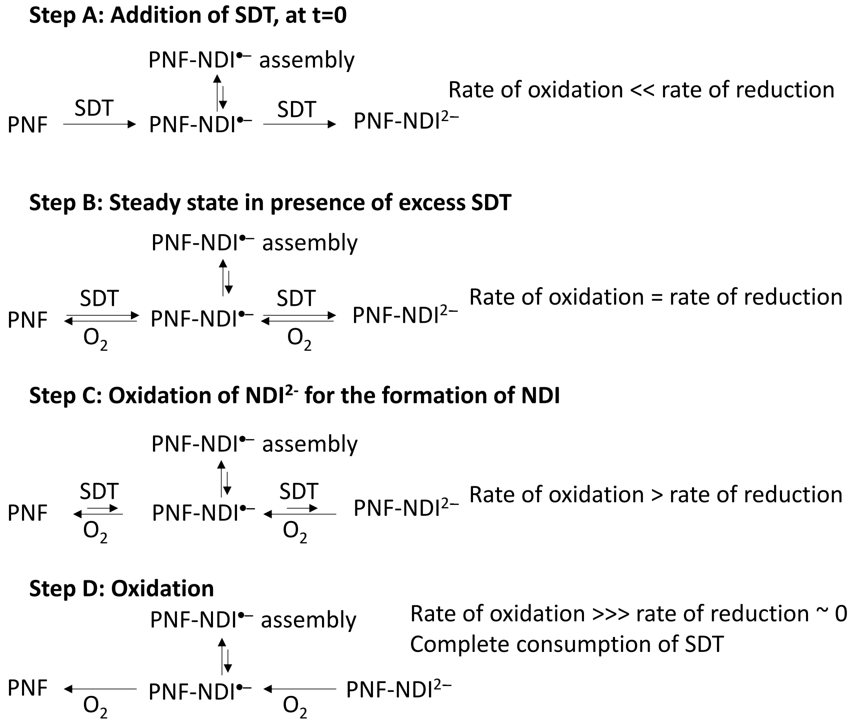

Step **A**, corresponds to the instantaneous formation of **PNF-NDI$^{2–}$**and **PNF-NDI$^{•–}$**from **PNF** on addition of excess eq. of SDT, where the rate of reduction is much faster than the rate of oxidation. **PNF- NDI$^{•–}$**due to its aggregation induced stabilization tends to be in **PNF-NDI$^{•–}$**assembled state (depicted by the DLS size data at t = 0 min) protecting it from spontaneous oxidation. Step **B** follows after step **A**, having a steady state of **PNF-NDI$^{•–}$**species, here **PNF-NDI$^{2–}$** gets oxidized to **PNF-NDI$^{•–}$**which in turn is reduced back to **PNF-NDI$^{2–}$** due to the presence of excess of SDT present in the solution (simultaneously any **PNF** formed is reduced back to **PNF-NDI$^{2–}$** and **PNF-NDI$^{•–}$**). Here the rate of oxidation equals to the rate of reduction and a steady concentration of **PNF-NDI$^{•–}$**(or its assembly, depicted by the DLS size data at t = 2 min. → 15 min.) is obtained. Since **PNF-NDI$^{•–}$**is stabilized in its assembled state, it has lower tendency to get oxidized to **PNF**, however, still this possibility cannot be ignored. The steady state is confirmed by minimal increase in size, ascribed to the formation of more **PNF-NDI$^{•–}$** species. Step **C** corresponds to the generation of **PNF-NDI$^{•–}$** from **PNF-NDI$^{2–}$**by controlled oxidation process where the rate of oxidation takes over the rate of reduction due to continuous consumption of oxygen, leading to an increase in the **PNF-NDI$^{•–}$** population as depicted by increased absorption and also the

formation of neutral **PNF** molecules which takes part in the formation of tape structures giving rise to slight increase in DLS size (depicted by the DLS size data at t = 45 min.). Finally at step **D**, the concentration of SDT becomes extremely low and the rate of reduction tends to zero and rate of oxidation dominates to convert all **PNF-NDI**$^{\bullet-}$ into active **PNF** molecules in a controlled rate that can undergo supramolecular polymerization into tape structures with increasing DLS size (depicted by the DLS size data at t = 50 min.).

Here, we expect the rate of dissolution of atmospheric oxygen into the solution, 1) remains constant throughout the cycle (system under equilibrium) and 2) is slower than the consumption of dissolved oxygen, as a result the rate of oxidation is mainly determined by amount of dissolved oxygen at t = 0 min. which is taken care while sample preparation. On addition of SDT, dissolved oxygen in the solution gets consumed. A lower amount of dissolved oxygen, keeps rate of oxidation low such that step B and step C will be longer. As a consequence, the kinetics of **PNF** formation are controlled by initial SDT concentration.

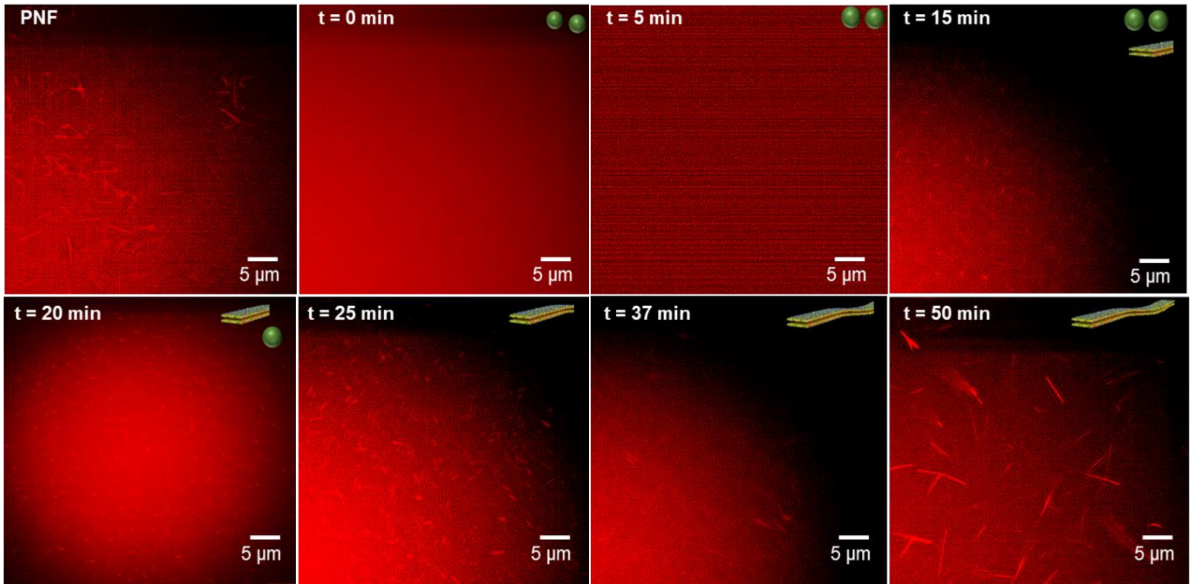

Supplementary Figure 19. Time-dependent structured illumination microscopic (SIM) images showing the disassembly of **PNF** tape structures after addition of 40 eq. SDT and the subsequent growth of the tapes over temporal oxidation (corresponding to the bright field images shown in Figure 5b). Inset schematics show the corresponding morphology present at each stages. The evolution of the fluorescent structures is due to the encapsulation of the Nile red dye inside the hydrophobic bilayer of the tapes. [**PNF**] = 5 x 10^{-5} M, CH_3CN/H_2O, (1:1 v/v). [Nile red] = 5 µM. λ_{exc} = 561 nm.

Supplementary Note 4: We have carried out the visualization process on reducing the sample in a vial and taken out the aliquots at different time point for the visualization. Here we could properly do the measurement of the sample both under bright field and SIM mode with minimum time lapse of 5 min. taking care of the focusing of the camera and fixing the sample in proper place. Hence after immediate reduction the first image captured at 5 min. time point.

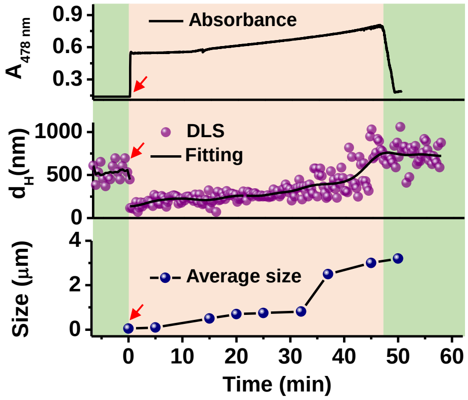

Supplementary Figure 20. Comparison of the average size of **PNF** assemblies obtained from the bright field microscopic images recorded at various time interval during the temporal oxidation of the reduced **PNF** with of 40 eq. SDT, with the time-dependent changes in absorbance and hydrodynamic diameter obtained from DLS. Arrow indicates the point of SDT injection. [**PNF**] = 5 x 10^{-5} M, CH_3CN/H_2O, (1:1 v/v).

Supplementary Note 5: Since the samples under SIM and bright field microscopic measurements are more exposed to air, the time kinetics for temporal oxidation and growth can have an error compared to DLS and absorbance kinetics although it is taken care by imaging the samples on petri dish under closed capped condition.

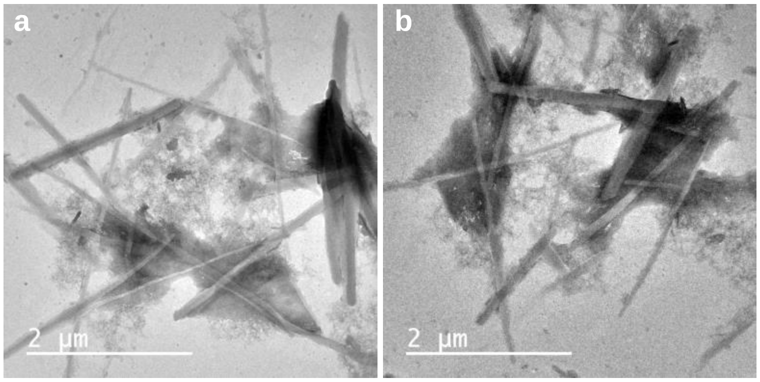

Supplementary Figure 21. **a.** and **b.** are the TEM images for the kinetically grown self-assembled structures using 40 eq. SDT. [**PNF**] = 5 x 10^{-5} M, CH_3CN/H_2O (1:1 v/v).

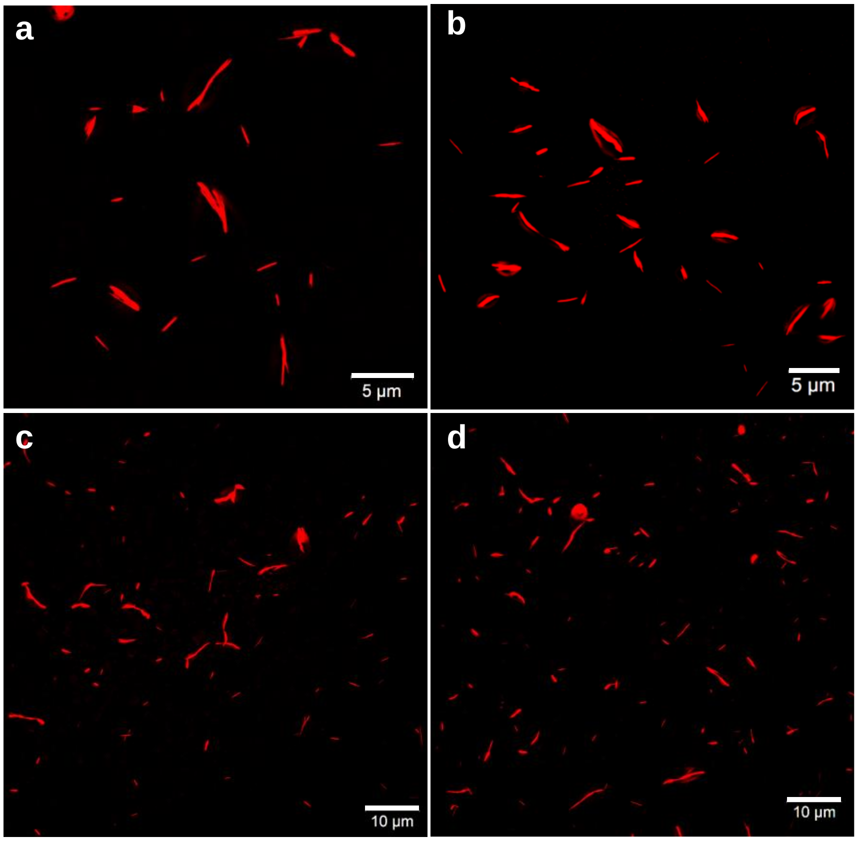

Supplementary Figure 22. Structured Illumination Microscopy (SIM) images for the instantaneously grown polydisperse **PNF** tape structures. **a**-**d.** are the Structured Illumination Microscopy (SIM) images for the instantaneously prepared tape structures of **PNF** loaded with Nile red and having a PDI of 1.24. [**PNF**] = 5 x 10^{-5} M, CH_3CN/H_2O, (1:1 v/v). Fluorescent probe: 5 µM Nile red. λ_{exc} = 561 nm.

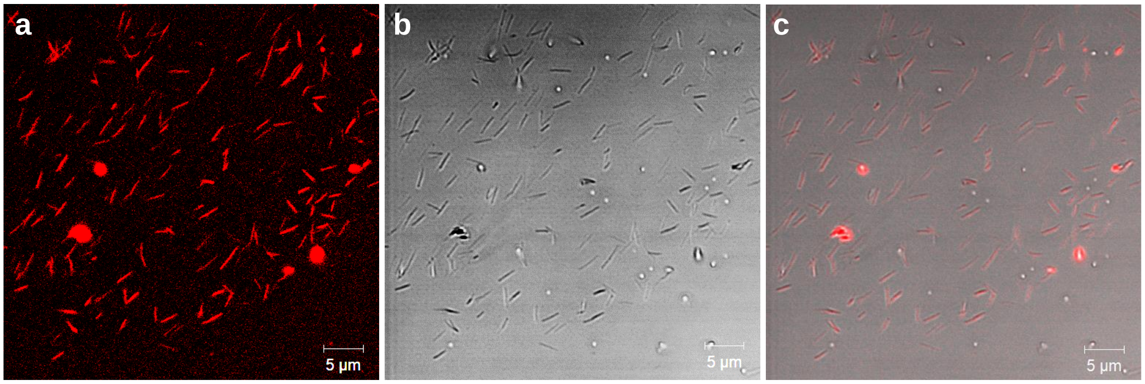

Supplementary Figure 23. CLSM image for the kinetically grown self-assembled tapes of **PNF** loaded with Nile red dye using 40 eq. SDT, showing a uniform distribution of Nile red probe dye throughout the structures after encapsulation in the hydrophobic bilayer. **a.** Fluorescence image, **b.** bright field image and **c.** merged image for the tape structures. [**PNF**] = 5 x 10^{-5} M, CH_3CN/H_2O, (1:1 v/v). Fluorescent probe: 5 µM Nile red. λ_{exc} = 561 nm.

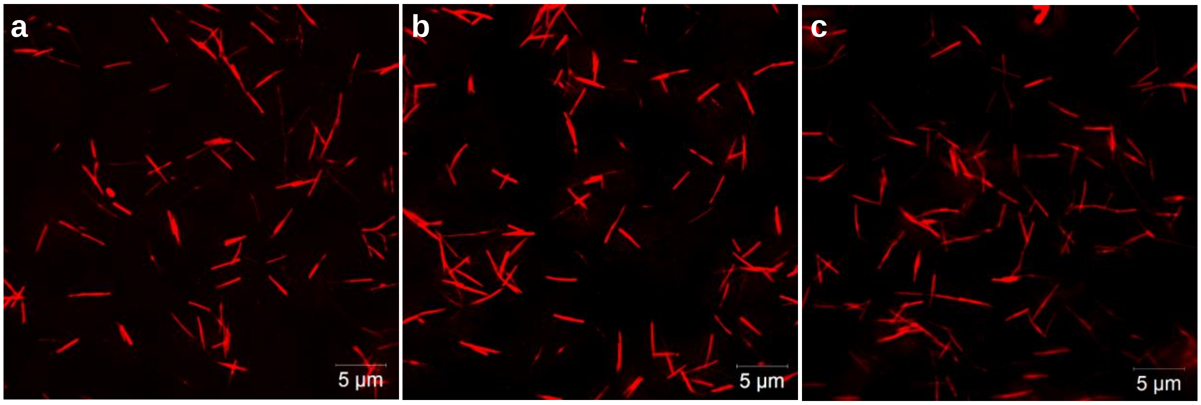

Supplementary Figure 24. Structured Illumination Microscopy (SIM) images for the monodisperse tape structures. **a**-**c**. are the SIM images of the self-assembled tapes of **PNF** loaded with Nile red dye, which are kinetically grown with 40 eq. of SDT. [**PNF**] = 5 x 10^{-5} M, CH_3CN/H_2O, (1:1 v/v). Fluorescent probe: 5 µM Nile red. λ_{exc} = 561 nm.

Supplementary Table 1. L_n, L_w, n and PDI data for instantaneous and kinetically grown self-assembled tape structures of **PNF** using various eq. of SDT.

Eq. SDT	L_n	L_w	n	PDI
0	1.83 µm	2.27 µm	473	1.24
20	2.03 µm	2.23 µm	472	1.10
40	3.00 µm	3.09 µm	350	1.03

L_w = weight-average length, L_n = number-average length, n = number of tapes counted, PDI = polydispersity index.

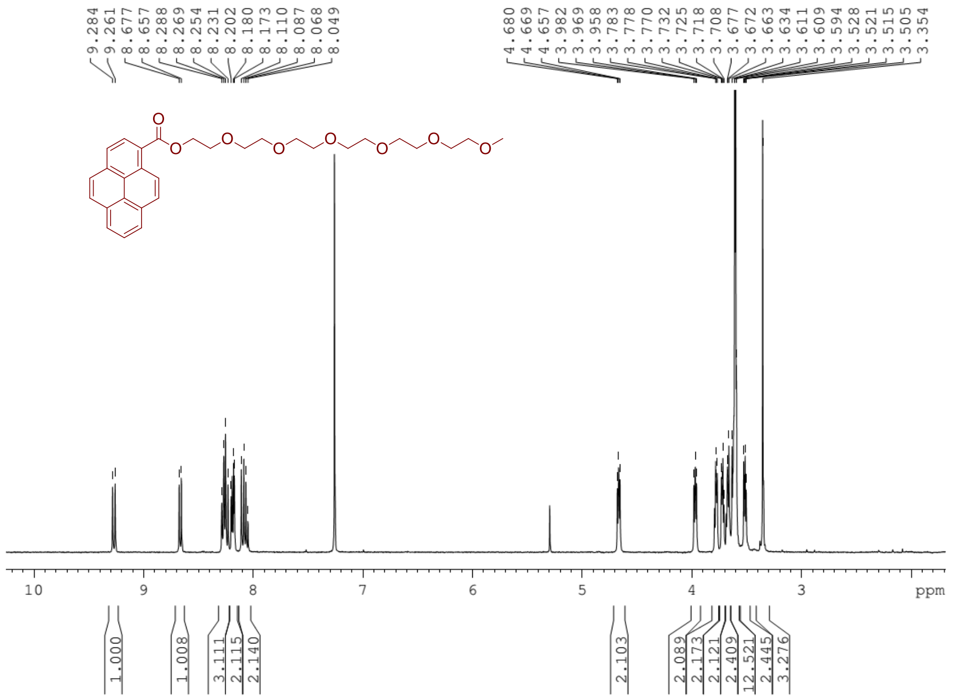

Supplementary Figure 25. ^{1}H NMR spectrum of molecule **Pyrene-HEG**. $CDCl_3$, 400 MHz, 298 K.

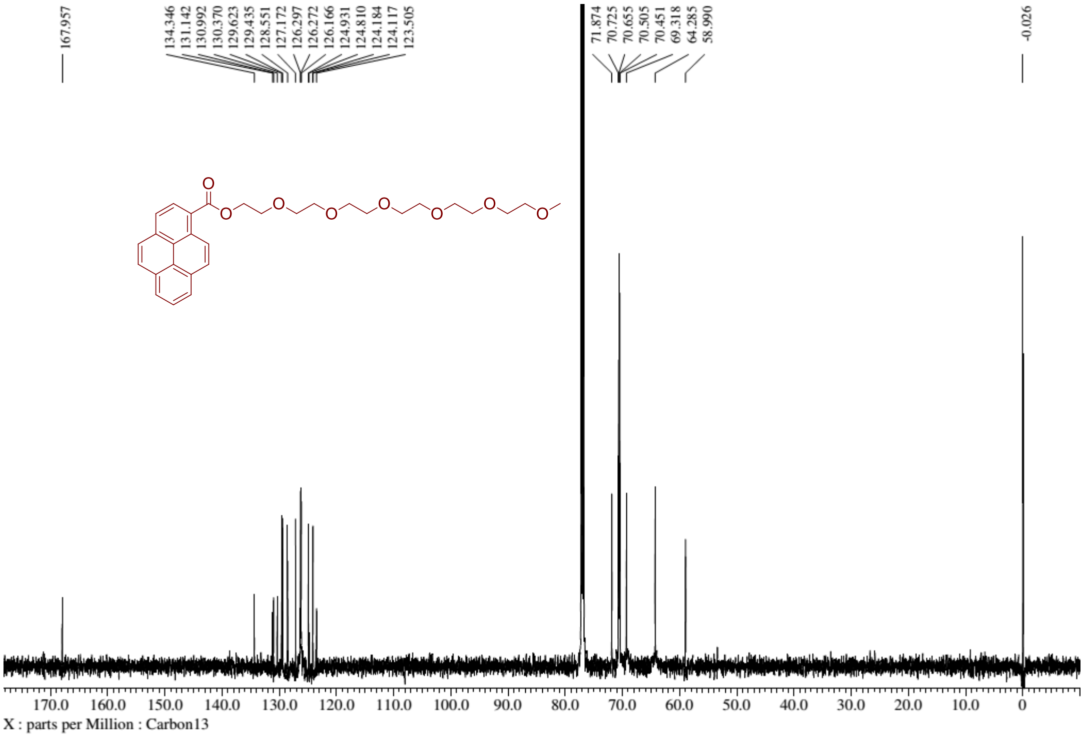

Supplementary Figure 26. ^{13}C NMR spectrum of molecule **Pyrene-HEG**. $CDCl_3$, 100 MHz, 298 K.

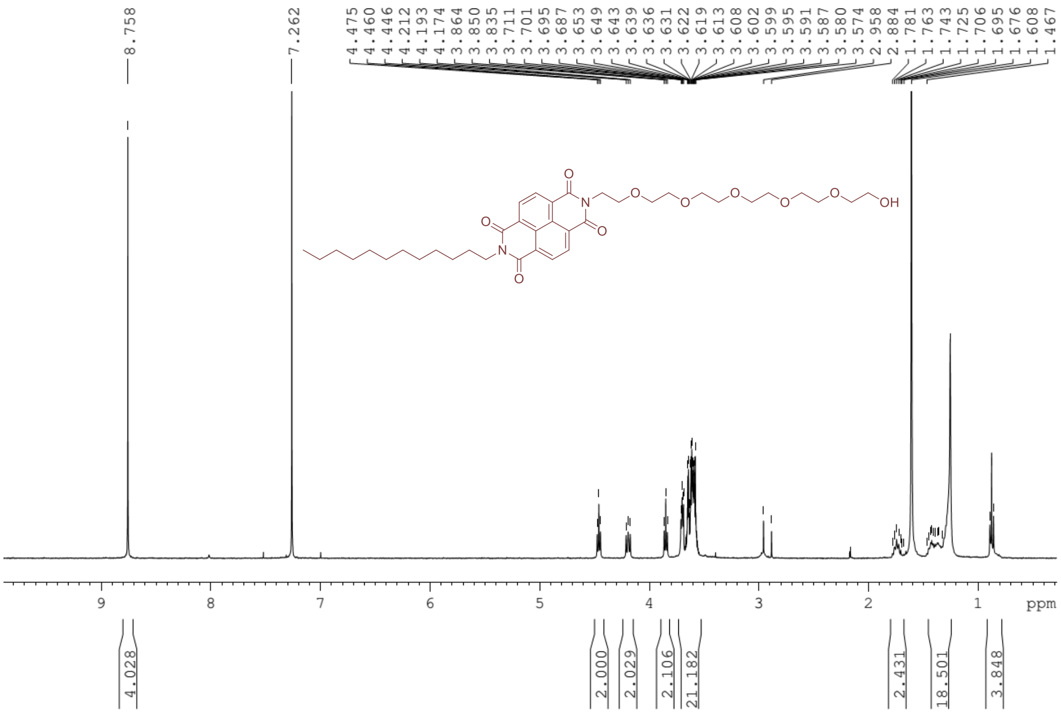

Supplementary Figure 27. ^{1}H NMR spectrum of molecule **3**. $CDCl_3$, 400 MHz, 298 K.

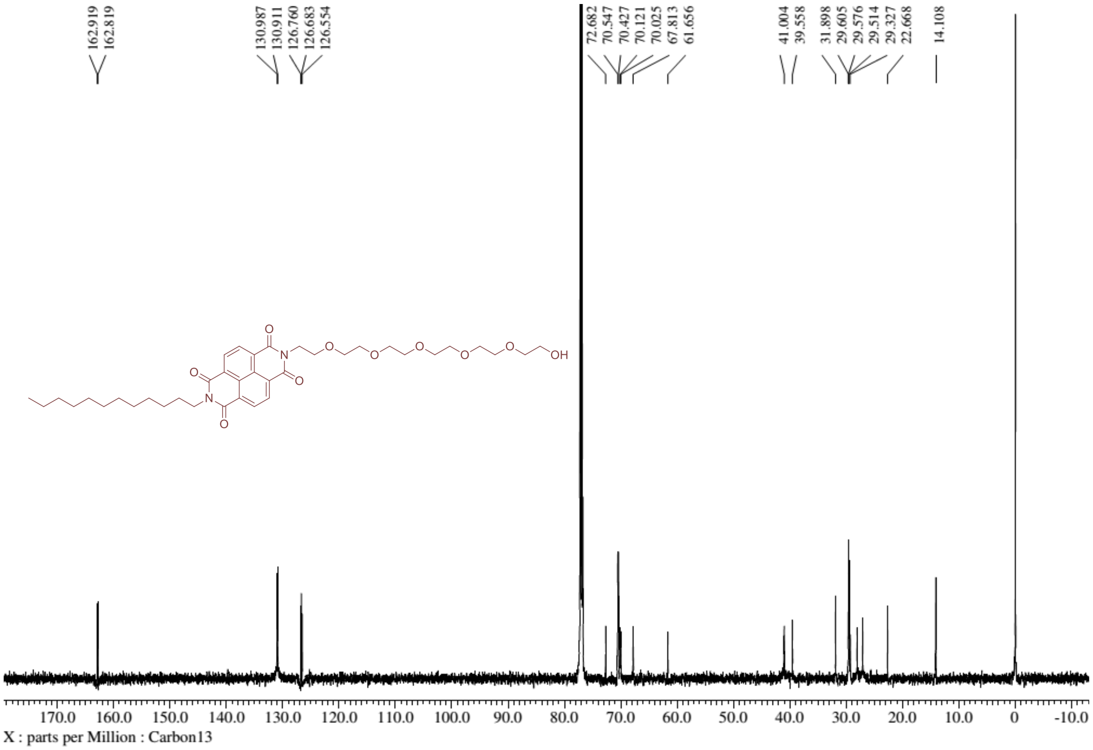

Supplementary Figure 28. ^{13}C NMR spectrum of molecule **3**. $CDCl_3$, 100 MHz, 298 K.

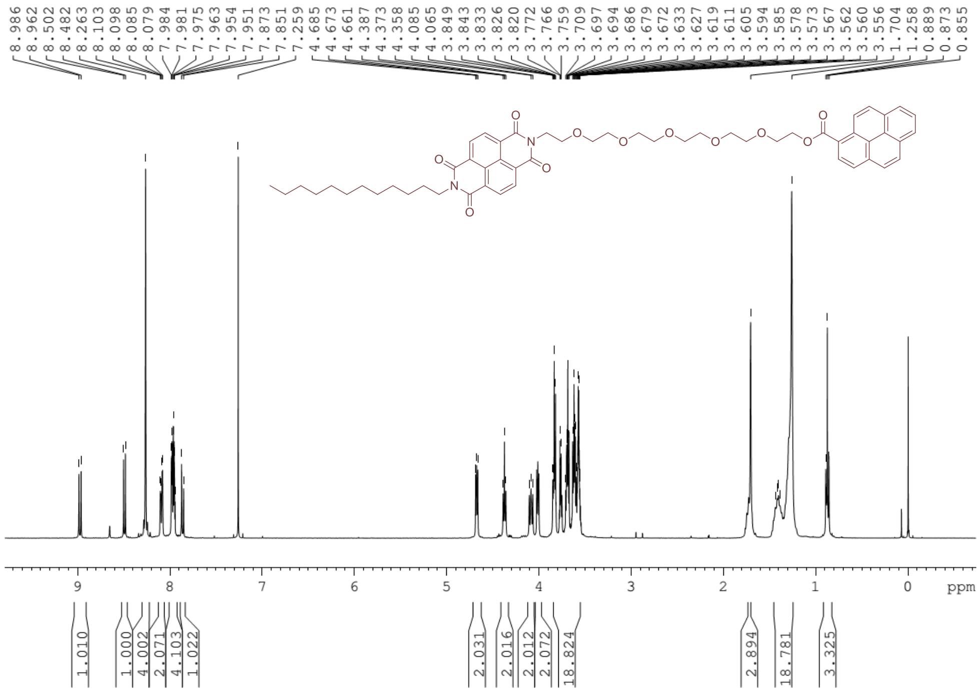

Supplementary Figure 29. ^{1}H NMR spectrum of molecule **PNF** foldamer. $CDCl_3$, 400 MHz, 298 K.

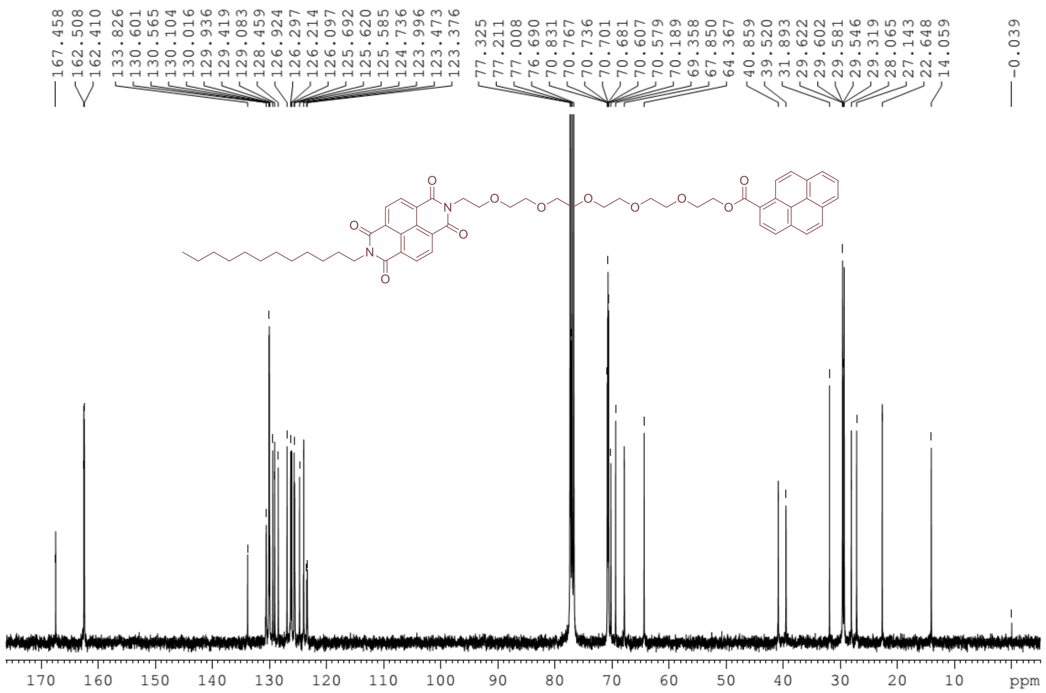

Supplementary Figure 30. ^{13}C NMR spectrum of molecule **PNF** foldamer. $CDCl_3$, 100 MHz, 298 K.

Supplementary References

1. Menger, F. M. & Zhang, H. Self-adhesion among phospholipid vesicles. *J. Am. Chem. Soc*. **128**, 1414-1415 (2006).

2. Israelachvili, J. N. Intermolecular and surface forces, 3rd ed.; Academic Press: London, 535-575 (1992).

3. Mitchel, J. & Ninham, B. W. Micelles, vesicles and microemulsions. *J. Chem. Soc. Faraday Trans*. 2. **77**, 601-629 (1981).

4. Antonietti, M. & Forster, S. Vesicles and liposomes: A self-assembly principle beyond lipids. *Adv. Mater.* **15**, 1323-1333 (2003).

5. Zhang, X., Chen, Z. & Würthner, F. Morphology control of fluorescent nanoaggregates by co-self-assembly of wedge- and dumbbell-shaped amphiphilic perylene bisimides. *J. Am. Chem. Soc.* **129**, 4886-4887 (2007).

6. Ramanathan, M., Shrestha, L. K., Mori, T., Ji, Q., Hill, J. P. & Ariga, K. Amphiphile nanoarchitectonics: from basic physical chemistry to advanced applications. *Phys. Chem. Chem. Phys*. **2013**, *15*, 10580-10611.

7. Tanford, C. Micelle shape and size. *J. Phys. Chem.* 1972*,* **76**, 3020-3024.
