# Supplementary material for: Transient dormant monomer states for supramolecular polymers with low dispersity
Source: Nat Commun. 2020 Aug 7;11:3967. doi: 10.1038/s41467-020-17799-w (PMC7415150; doi:10.1038/s41467-020-17799-w)
Supplement: Supplementary file 2 — Description of Additional Supplementary Files [file 41467_2020_17799_MOESM2_ESM.pdf]

Description of Additional Supplementary Files

File Name: Supplementary Movie 1
Description: Real time visualization of the reduction induced disassembly process of the one-dimensional tapes (PNF) to spherical aggregates ($PNF\text{-}NDI^{\bullet-}$) under bright field microscopy, upon addition of 40 eq. SDT. [PNF] = 5×10^{-5} M, CH_3CN/H_2O (1:1 v/v). pH = 8 buffer.

File Name: Supplementary Movie 2
Description: Real time visualization of the reduction induced disassembly process of the one-dimensional tapes (PNF) to spherical aggregates ($PNF\text{-}NDI^{\bullet-}$) under bright field microscopy, upon addition of 40 eq. SDT. [PNF] = 5×10^{-5} M, CH_3CN/H_2O (1:1 v/v). pH = 8 buffer.
